# *In Vitro* Characterization of Echinomycin Biosynthesis: Formation and Hydroxylation of L-Tryptophanyl-S-Enzyme and Oxidation of (2*S*,3*S*) β-Hydroxytryptophan

**DOI:** 10.1371/journal.pone.0056772

**Published:** 2013-02-21

**Authors:** Chen Zhang, Lingxin Kong, Qian Liu, Xuan Lei, Tao Zhu, Jun Yin, Birun Lin, Zixin Deng, Delin You

**Affiliations:** 1 State Key Laboratory of Microbial Metabolism and School of Life Sciences and Biotechnology, Shanghai Jiao Tong University, Shanghai, People’s Republic of China; 2 Institute of Plant Protection, Guangdong Academy of Agricultural Sciences, Guangzhou, People’s Republic of China; National Centre for Cell Science, India

## Abstract

Quinoxaline-2-carboxylic acid (QXC) and 3-hydroxyquinaldic acid (HQA) feature in quinomycin family and confer anticancer activity. In light of the significant potency against cancer, the biosynthetic gene clusters have been reported from many different *Streptomyces* strains, and the biosynthetic pathway were proposed mainly based on the *in vivo* feeding experiment with isotope labeled putative intermediates. Herein we report another gene cluster from *Streptomyces griseovariabilis* subsp. bandungensis subsp. nov responsible for the biosynthesis of echinomycin (a member of quinomycin family, also named quinomycin A) and presented *in vitro* evidence to corroborate the previous hypothesis on QXC biosynthesis, showing that only with the assistance of a MbtH-like protein Qui5, did the didomain NRPS protein (Qui18) perform the loading of a L-tryptophan onto its own PCP domain. Particularly, it was found that Qui5 and Qui18 subunits form a functional tetramer through size exclusion chromatography. The subsequent hydroxylation on β-carbon of the loaded L-tryptophan proved *in vitro* to be completed by cytochrome P450-dependent hydroxylase Qui15. Importantly, only the Qui18 loaded L-tryptophan can be hydroxylated by Qui15 and the enzyme was inactive on free L-tryptophan. Additionally, the chemically synthesized (2*S*,3*S*) β-hydroxytryptophan was detected to be converted by the tryptophan 2,3-dioxygenase Qui17 through LC-MS, which enriched our previous knowledge that tryptophan 2,3-dioxygenase nearly exclusively acted on L-tryptophan and 6-fluoro-tryptophan.

## Introduction

**Figure 1:**
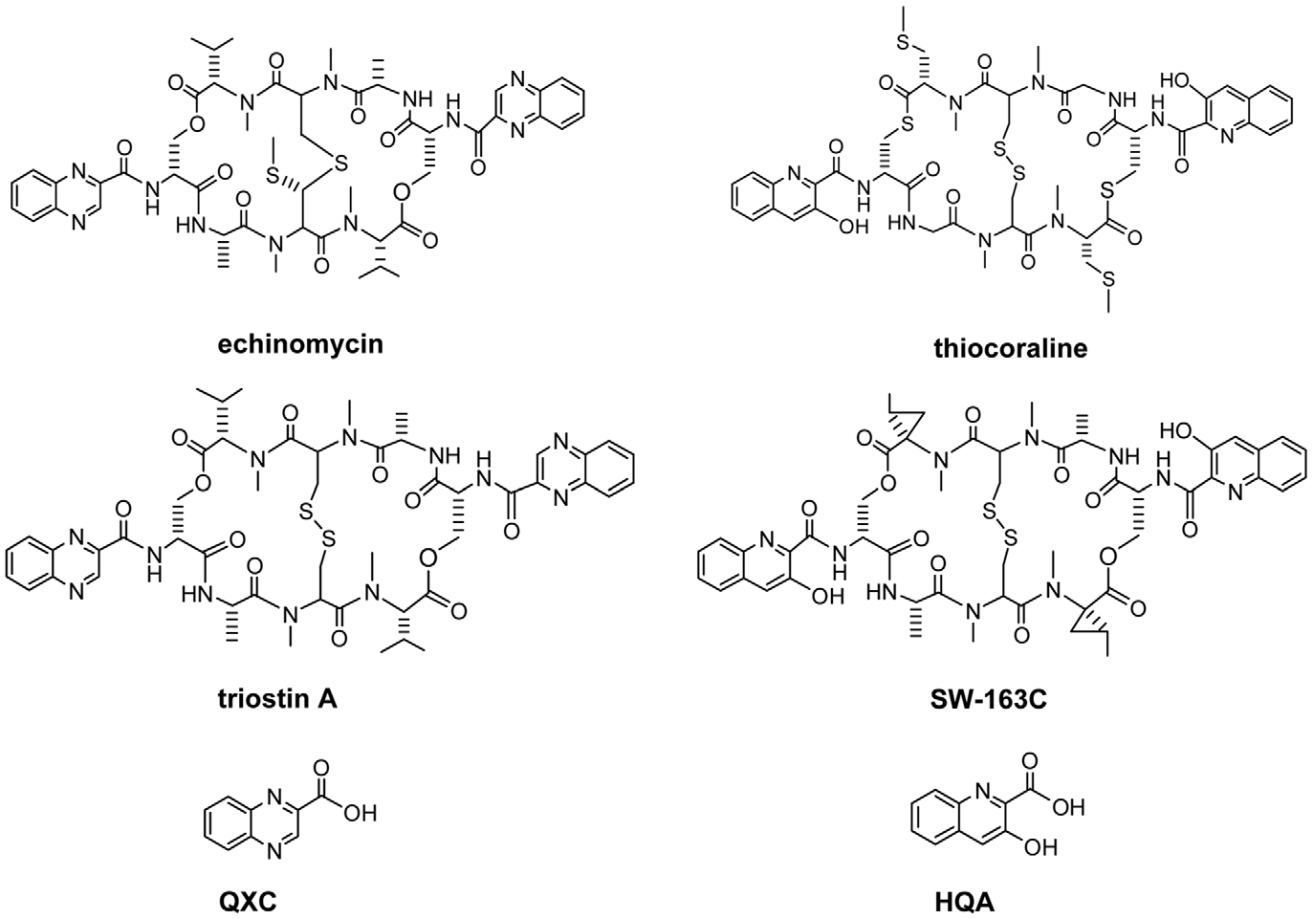
Structures of echinomycin, thiocoraline,triostin A, SW-163C, QXC and HQA. QXC: quinoxaline-2-carboxylic acid; HQA: 3-hydroxyquinaldic acid. Echinomycin and triostin A have QXC as their aromatic precursor during biosynthesis while thiocoraline and SW-163C have HQA as the aromatic precursor.

Quinomycin antibiotics are composed of the nonribosomal peptide (NRP) backbone and a pair of identical quinoxaline or 3-hydroxyquinaldic rings (Figure 1), which originates respectively from the aromatic precursor of quinoxaline-2-carboxylic acid (QXC) and 3-hydroxyquinaldic acid (HQA) [1] (Figure 1). The two aromatic compounds are structural analogs, crucial for the anticancer potency of quinomycin, including echinomycin (quinomycin A), triostin, SW-163 and thiocoraline via the intercalation of their QXC (for echinomycin and triostin) or HQA (for SW-163 and thiocoraline) into DNA base pairs to form a sandwiching structure so that both DNA replication and transcription are inhibited, despite the difference in the insertion hotspot on DNA which are determined by the amino acid residues in the NRP backbones [2], [3], [4]. The biosynthetic gene clusters for echinomycin [5], [6] and triostin [7] indicated that the QXC and HQA biosynthesis genes have high homology among some counterparts especially in the enzymes assigned to first several steps of QXC and HQA biosynthesis pathway. In detail for the aromatic precursor biosynthesis, at first, with the help of an MbtH-like protein, L-tryptophan is loaded onto a NRPS protein which consists of both adenylation (A) and PCP (T) domain, the assistance of the MbtH-like protein in the activity of the NRPS protein in echinomycin and triostin A biosynthesis was proposed by Kenji Watanabe, yet unsubstantiated [6], [7]. A domain activates L-tryptophan by adenylation and subsequently catalyzes the formation of a thioester bond between the carbonyl group of adenylated tryptophan and the thiol group of phosphopantetheine on PCP domain. The loaded tryptophan is hydroxylated at β-carbon by a cytochrome P450 superfamily member to produce (2*S*,3*S*) β-hydroxytryptophanyl-S-PCP. The hydroxylated tryptophan is then released by a type II thioesterase to present (2*S*,3*S*) β-hydroxytryptophan. The free hydroxytryptophan undergoes a oxidative indole-ring opening mediated by a member of tryptophan 2,3-dioxygenase superfamily to form (2*S*,3*R*) *N*-formyl-β-hydroxykynurenine, which is hydrolyzed by a hydrolase to yield (2*S*,3*R*) β-hydroxykynurenine. For the QXC pathway, the subsequent reaction is an oxidative rearrangement fulfilled by a FAD-dependent oxidoreductase, giving rise to *N*-(2′-aminophenyl)-β-hydroxyaspartic acid, the intermediate was converted to *N*-(2′-aminophenyl)-β-ketoaspartic acid by a dehydrogenase. The ensuing three steps of decarboxylation, cyclization and oxidative aromatization reaction occur spontaneously to produce the last aromatic precursor QXC [8]. For the HQA pathway, the amino group on the α-carbon of (2*S*,3*R*) β-hydroxykynurenine is converted by an aminotransferase to form a carbonyl group, which is linked to the amino group steming from the nitrogen atom of L-tryptophan, resulting in the formation of another six-membered ring neighboring the benzyl ring, finally, it is reduced to HQA [9].

Although the hypothesis for the QXC or HQA biosynthetic pathway has been proposed for several years, as yet, very few definite *in vitro* data exist to support them, leaving behind some uncertainties. Here we report the cloning and sequencing of the echinomycin biosynthesis gene cluster from *Streptomyces griseovariabilis* subsp. bandungensis subsp. nov, and demonstrate that MbtH-like protein and a NRPS protein form complex to mediate β-hydroxylation of L-tryptophan by cytochrome P450 hydroxylase, and a tryptophan 2,3-dioxygenase acting on (2*S*,3*S*) β-hydroxytryptophan in biosynthetic pathway of QXC.

## Results and Discussion

### Cloning of Echinomycin Biosynthesis Gene Cluster from *S. griseovariabilis* subsp. bandungensis subsp. nov

**Figure 2:**
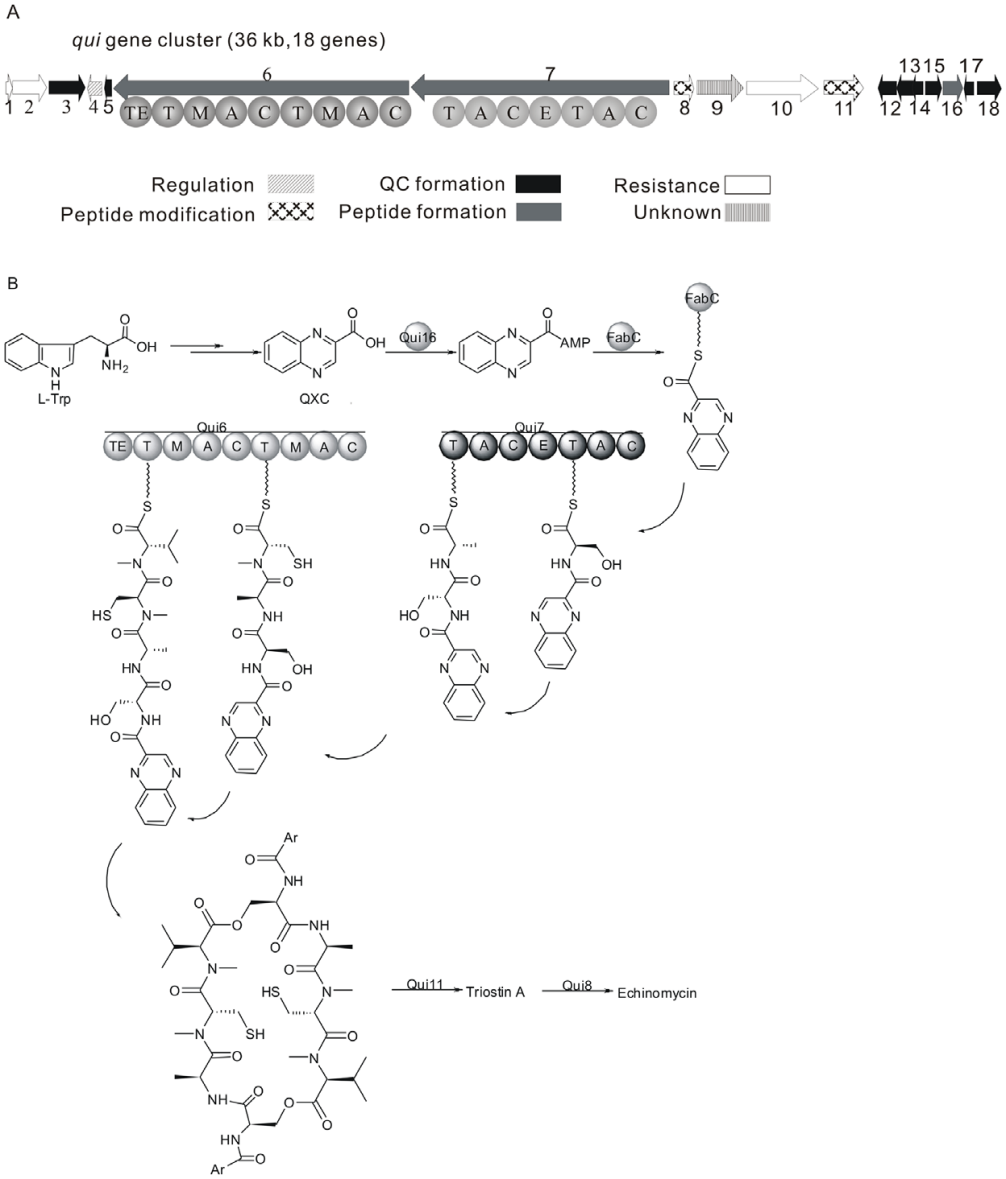
Organization of echinomycin biosynthetic gene cluster and echinomycin biosynthetic pathway in *S. griseovariabilis*. (A) Scheme of the biosynthetic gene cluster of echinomycin in *S. griseovariabilis*. The transcription direction of each gene was indicated by arrow. *qui6* and *qui7* are NRPS genes encoding the NRPS responsible for biosynthesis of the NRP backbone of echinomycin. To clarify the domains contained in Qui6 and Qui7, the scheme of the protein products encoded by *qui6* and *qui7* were situated rightly below the corresponding genes. Since the transcription directions of both *qui6* and *qui7* were reversed, their protein products were also exhibited inversely. From N terminal to C terminal, the domains contained were C, A, M, T, C, A, M, T, TE in Qui6 and C, A, T, E, C, A, T in Qui7. C: condensation-domain; A: adenylation-domain; T: peptidyl carrier protein-domain; M: methylation-domain; E: epimerase-domain; TE: thioesterase-domain. (B) Echinomycin biosynthetic pathway in *S. griseovariabilis*. FabC was an ACP engaged in fatty acid biosynthesis and its encoding gene was outside the gene cluster.

The adenylation domain of QXC for echinomycin biosynthesis shares high homology with both *ecm* gene cluster from *Streptomyces lasaliensis* and *trs* gene cluster from *Streptomyces triostinicus* [10]. The conserved regions of TrsA QXC adenylation domain was used to design primers for screening the echinomycin biosynthesis gene cluster in genomic library of *S. griseovariabilis* subsp. bandungensis subsp. nov. A PCR product of around 600 bp would be displayed by the positive sample, as expected. Through a pool- and-split PCR approach with the chosen primers, two clones respectively containing fosmid of A111 and K311 were picked out from 14 96-well plates (Figure S1). Randomly subcloned into pBlueScript II SK plus for sequencing, K311 was found to contain all echinomycin biosynthesis related genes found in A311. K311 was sequenced (NCBI accession number: JN852959) and analyzed with FramePlot and 18 genes were predicted to be involved in echinomycin biosynthesis (Figure 2). The arrangement and orientation of the 18 genes were exactly the same as *trs* gene cluster. Besides, all the 18 genes could found their highly homologous counterparts in *trs* gene cluster with the nucleotide identity ranging from 93% to 98%. In contrast, the distinction from *ecm* gene cluster was far more obvious, on one hand, *qui1* and *qui2* didn’t have corresponding homologs, that is, only 16 remaining genes had counterparts in *ecm* gene cluser, on the other hand, the arrangement was different from that of *ecm* gene cluser and the identity was much lower than those of *trs* gene cluster, averaging lower than 80%. Particularly, the alignment with the tryptophan 2,3-dioxygenase of *ecm* gene cluster didn’t even show remarkable similarity at nucleotide level, forming an obvious contrast to the result of the other 15 genes, among which the lowest identity was above 70% (Table. 1).

**Figure 3:**
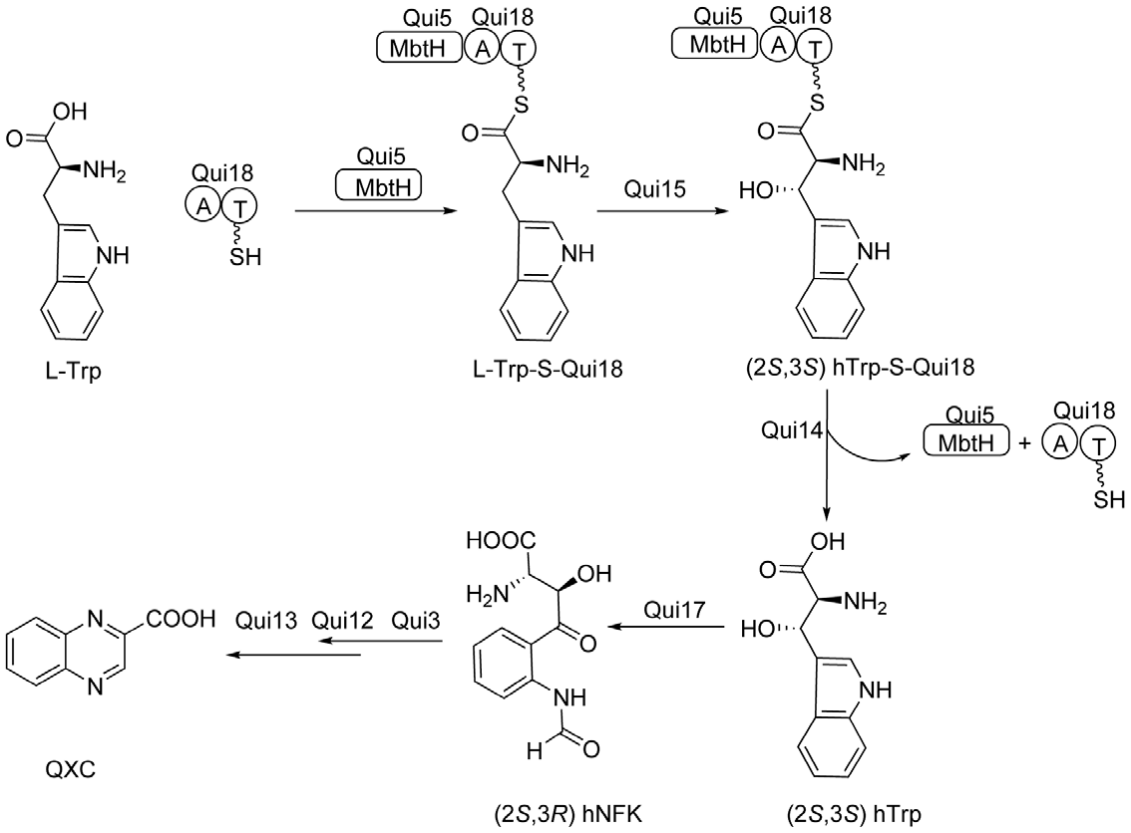
Proposed biosynthetic pathway for QXC produced by ***S. griseovariabilis*** **.** The biosynthesis of QXC is a part of that of echinomycin. L-Trp: L-tryptophan; L-Trp-S-Qui18: L-tryptophanyl-S-Qui18; (2*S*,3*S*) hTrp-S-Qui18: (2*S*,3*S*) β-hydroxytryptophanyl-S-Qui18; (2*S*,3*S*) hTrp: (2*S*,3*S*) β-hydroxytryptophan; (2*S*,3*R*) hNFK: (2*S*,3*R*) *N*-formyl-β-hydroxykynurenine; QXC: quinoxaline-2-carboxylic acid.

Referring to the result from *ecm* gene cluser and *trs* gene cluser, the pathway in *S. griseovariabilis* subsp. bandungensis subsp. nov was deduced (Figure 2B and 3). Seven genes are responsible for the biosynthesis of the aromatic precursor QXC. They are *qui18*, *qui15*, *qui14*, *qui17*, *qui3*, *qui12* and *qui13* in order and respectively homologous to *trsR*, *trsB*, *trsQ*, *trsC*, *trsF*, *trsO* and *tr*s*P* (Table 1). As the beginning precursor, L-tryptophan is loaded onto a NRPS protein Qui18, which contained both adenylation (A) and PCP (T) domain. A domain activated L-tryptophan by adenylation and subsequently catalyzed the formation of a thioester bond between the carbonyl group of adenylated tryptophan and the thiol group of phosphopantetheine on PCP domain and thereby loading tryptophan onto the PCP domain of Qui18 to form tryptophanyl-S-Qui18; then under the catalysis of the other proteins, the loaded complex was in turn converted to (2*S*,3*S*) β-hydroxytryptophanyl-S-Qui18, (2*S*,3*S*) β-hydroxytryptophan, (2*S*,3*R*) *N*-formyl-β-hydroxykynurenine and (2*S*,3*R*) β-hydroxykynurenine *N*-(2′-aminophenyl)- β-hydroxyaspartic acid and *N*-(2′-aminophenyl)-β-ketoaspartic acid which underwent three spontaneous reactions to give rise to QXC (Figure 3).

**Table 1:** Deduced functions of the ORFs in the echinomycin biosynthetic gene cluster from *S. griseovariabilis* and nucleotide sequence alignment with echinomycin biosynthetic gene cluster from *S. lasaliensis* and triostin A biosynthetic gene cluster from *S. triostinicus*.

Gene	Amino acids	Deduced function	*S. lasaliensis* homolog	*S. triostinicus* homolog
qui1 (partial)	50	ABC transporter	−	trsD (95%)
qui2	275	ABC transporter	−	trsE (96%)
qui3	402	Esterase	ecm14 (75%)	trsF (95%)
qui4	177	DNA-binding response regulator	ecm9 (81%)	trsG (98%)
qui5	71	MbtH-like protein	ecm8 (86%)	trsH (97%)
qui6	3146	Non-ribosomal peptide synthetase	ecm7 (81%)	trsI (93%)
qui7	2665	Non-ribosomal peptide synthetase	ecm6 (86%)	trsJ (95%)
qui8	233	SAM-dependent methyltransferase	ecm18 (75%)	trsK (95%)
qui9	427	Hypothetical protein	Not annotated (75%)	trsL (94%)
qui10	793	UvrA-like protein	ecm16 (87%)	trsM (97%)
qui11	313	Thioredoxin reductase	ecm17 (77%)	trsN (94%)
qui12	463	FAD-dependent oxidoreductase	ecm4 (79%)	trsO (95%)
qui13	363	Isopropylmalate dehydrogenase	ecm3 (82%)	trsP (94%)
qui14	249	Thioesterase	ecm2 (77%)	trsQ (97%)
qui15	402	Cytochrome P450 hydroxylase	ecm12 (76%)	trsB (97%)
qui16	528	AMP-binding ligase	ecm1 (83%)	trsA (95%)
qui17	275	Tryptophan 2,3-dioxygenase	ecm11 (very low)	trsC (95%)
qui18	611	Non-ribosomal peptide synthetase	ecm13 (73%)	trsR (96%)

The sequence of *qui1* wasn’t complete, so it is labeled ‘partial’. The last two columns reveals the identity at nucleotide level of genes in the cluster of *S. griseovariabilis* to the homologs in *S. lasaliensis* and *S. triostinicus.* The number in the bracket denotes the identity; ‘−’ means no homologous gene was found, ‘very low’ means the putative homologous gene doesn’t have obvious identity, but is still considered homologous because of the amino acid identity of 46%.

The aromatic precursor was then adenylated by the trsA homologous protein Qui16 and loaded to FabC, which was in fact a free-standing ACP involved in the primary metabolism of fatty acid biosynthesis and whose encoding gene lay out of the gene cluster [10]. The biosynthesis of NRP backbone was encompassed by two NRPS bi-modular proteins Qui7 and Qui6, homolog of TrsJ and TrsI. Qui7 was responsible for the incorporation of Ser and Ala, Qui6 for Cys and Val. After the incorporation of Ser by Qui7, the adenylated quinoxaline-2-carboxylic aromatic precursor was linked to its amino group by the first C domain of Qui7. Besides, both the two modules of Qui6 had *N*-methylation domain downstream of the corresponding T domains, that is why Cys and Val in the NRP backbone are methylated at their α-amino groups. The aromatic precursor-coupled NRP was set free from the last PCP of Qui6 (homolog of TrsI) by the downstream TE domain via cyclodimerization, connecting two identical oligopeptides head to tail in the form of macrolide with the hydroxyl group of Ser bound to α-carboxyl group of *N*-methyl Val of the other chain. Two thiol groups of *N*-methyl Cys in the macrolide were linked together by Qui11 (homolog of TrsC) to form a disulfide bond, which produced triostin. Qui8 (homolog of TrsK), a SAM-dependent methyl transferase could convert triostin A to echinomycin by methylation and spontaneous rearrangement, transforming disulfide bond into thioacetal bond [6] (Figure 2B), but in *S. triostinicus*, the fermentation product was detected to contain mainly triostin A and minor echinomycin, in contrast, *S. griseovariabilis* was detected to produce echinomycin without triostin A. The difference seemed not to be attributable to the activity of TrsK, since its high identity to Qui8. More plausibly, it might be caused by substitution of two ‘AT’ pairs in the promoter of *qui8* with ‘GC’ pairs in that of *trsK* thereby attenuating the promoter of *trsK* and undermining the production of TrsK, and the scarcity of TrsK would cause the accumulation of triostin A for converting a minority of triostin A.

One putative regulation gene *qui4* (homolog of *trsG*) was detected in the gene cluster and predicted to be DNA-binding response regulator. Three genes *qui1*, *qui2* and *qui10* (homolog of *trsD*, *trsE* and *trsM*) were assigned to confer self-resistance, the mechanism of which seems complex, for two of them were categorized as ABC transporter, and their homologs were only found in the cluster of triostinicus. The other self-resistance gene was identified as UvrA-like protein, and its homolog is present in both *trs* and *ecm* gene clusters. Although UvrA was known to be ATP-dependent DNA binding protein involved in excision repair of DNA, the self-resistance mechanism might be quite different from that of excision repair, since some UvrA-like proteins were confirmed to be ATP-dependent bacterial antiporters, such as DrrA, a daunorubicin resistance protein [11], [12]. Hence, how the self-resistance of echinomycin was realized remains unresolved.

### Determination of Substrate Specificity of Qui18

Bioinformatic analysis demonstrated that Qui18 was an A-PCP two-domain NRPS protein, yet the database (http://nrps.igs.umaryland.edu/nrps/2metdb/) failed to provide a definite prediction on which amino acid the A domain of Qui18 would recognize and load. Although the existent hypothesis on echinomycin biosynthesis favored L-tryptophan as the substrate and postulated the assistance of MbtH-like protein in the loading process, there hasn’t yet been powerful evidence to support it. Besides, the present papers reporting the type of reaction catalyzed by A-PCP two-domain NRPS protein in association with MbtH-like protein revealed the loading of tyrosine in the biosynthesis of novobiocin, clorobiocin, simocyclinone D8, pacidamycin and vancomycin [13], [14], [15]. Also, MbtH-like protein was demonstrate to be not necessarily indispensable in some amino acid loading reaction catalyzed by NRPS protein [16]. Therefore, we tried to clarify here what amino acid would be loaded by Qui18 and whether the MbtH-like protein Qui5 would be necessary for the loading reaction.

**Figure 4:**
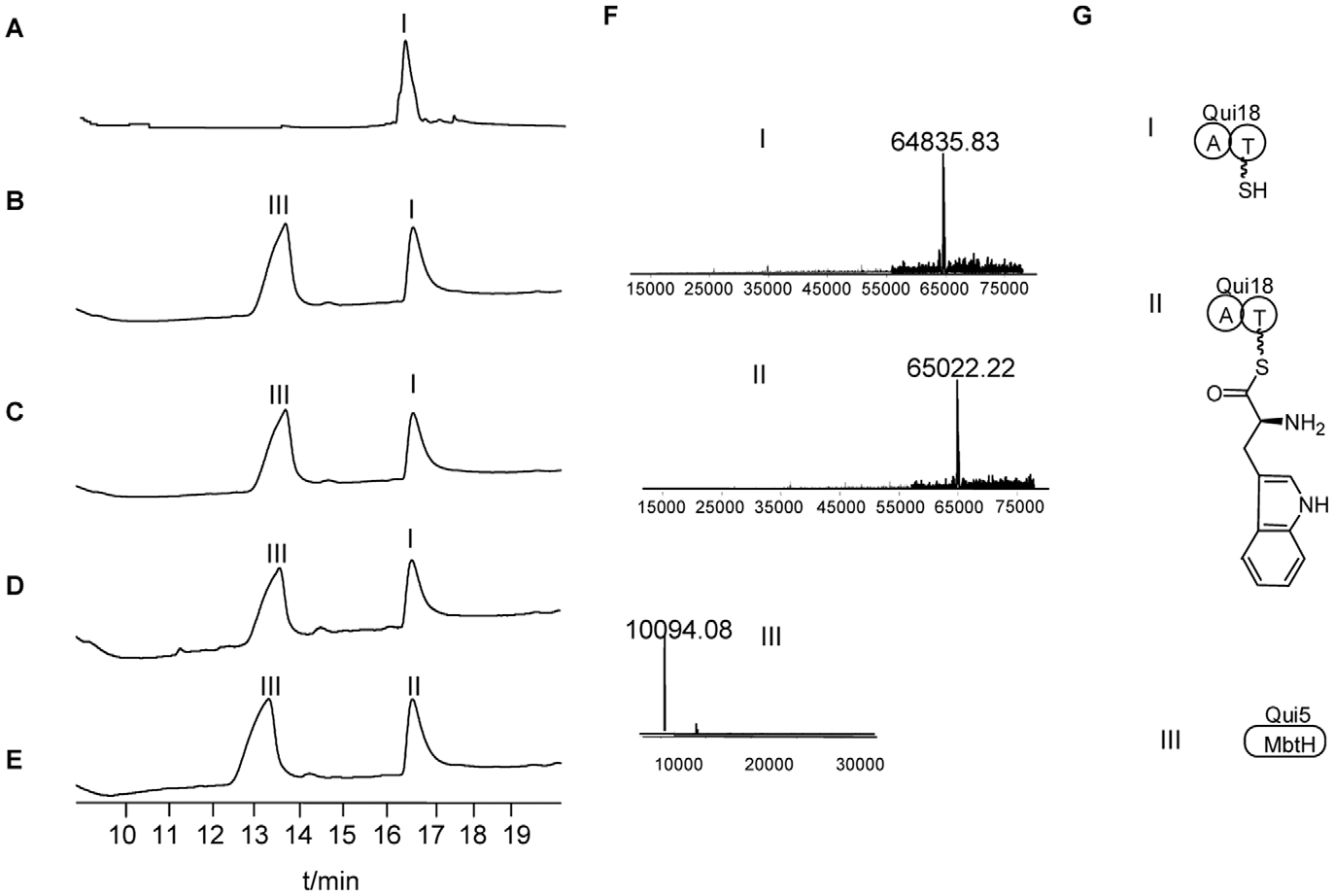
Q-TOF-MS analysis of the amino acid loading activity of Qui18. (A) Holo-Qui18 was used as the enzyme, L-tryptophan was tested as substrate; (B) The same as (A) except Qui5 was supplemented (C) Coexpression of Qui5 and holo-Qui18 was used as the enzyme, D-tryptophan was tested as substrate; (D) The same as (C) except the tested substrate was L-phenylalanine; (E) The same as (C) except the tested substrate was L-tryptophan; (F) MS result of the peak I, II and III, which respectively correspond to holo-Qui18, L-tryptophan loaded holo-Qui18 and Qui5; (G) Illustration of the protein molecule corresponding to I, II and III. The molecular weight change from I to II was so small relatively to the molecular weight of I that the retention time for I and II were nearly the same.

During the expression and purification of related proteins, we found that much more soluble Qui18 would be acquired by coexpression of both *qui5* and *qui18* than by the expressing *qui18* alone. SDS-PAGE showed a little higher amount of Qui18 in the 100-fold diluted coexpression sample than the undiluted solely expressed sample (Figure S2). Through coexpression, the two proteins Qui5 and Qui18 could be copurified through Ni column affinity chromatography, since both the two genes in pJTU5906 carried His_6_ tag (Figure S2). It was noteworthy that the copurified product was concentrated with Millipore Amicon Ultra Centrifugal Filter Units 50 kD, meaning that proteins smaller than 50 kD would be lost through centrifugation. Although Qui5 was only about 10 kD, it could still be detected through both SDS-PAGE and mass spectrometry, which suggested that Qui5 might bind to Qui18, which is big enough for the retention by Millipore Amicon Ultra Centrifugal Filter Units whereby the tightly bound Qui5 could be reserved and detected together with Qui18. More direct evidence to show the binding of Qui5 to Qui18 was provided by size exclusion chromatography which showed a peak corresponding to the mass weight of around 160 kD (Figure S3), and the SDS-PAGE analysis about the fraction showed the existence of Qui5 and Qui18 at about an equal amount (data not shown), hence it can be concluded that two subunits of Qui5 and two subunits of Qui18 form a hetero-tetramer. Before doing any enzymatic study on Qui18, we must mature it by phosphopantetheinylating its PCP domain, which was realized by coexpression with *sfp* gene (*Bacillus subtills* PPTase gene expressed from pSV20) during fermentation. Both theoretical computation and ESI-QTOF-MS indicated the molecular weight of apo and holo-Qui18 was respectively 64,496 and 64,836 Da, which was a +340 mass shift consistent with 4′-phosphopantetheine (4′-PP) cofactor covalently attached to the conserved Ser residue of apo-Qui18. The *in vivo* phosphopantetheinylation efficiency was so high that the protein sample purified from pSV20 containing strain gave a peak at 16.8 min, having an ion signal of m/z at 64,836 Da and the signal of m/z at 64,496 Da was utterly undetected by ESI-QTOF-MS (data not shown). The coexpression sample of *qui5* and *qui18* or *qui5*, *qui18* and *sfp* gave another peak at 13.3 min, having a corresponding ion signal of m/z at 10,094 Da which was assigned to Qui5, and not surprisingly, no signal of Sfp was detected since Sfp didn’t carry a His_6_ tag. Given that the coexpression of Qui5 and Qui18 and Sfp could produce Qui18 at much higher concentration and the copurification of Qui5, Qui18 and Sfp could also guarantee the existence of mature Qui18 and Qui5, presumably in a complex form, the coexpression product of the three genes was directly used for the *in vitro* assay. After incubation for 2 hours, the sample in which coexpression product of Qui5 and holo-Qui18 were used with L-tryptophan added in showed a mass shift of +186, giving an ion signal of m/z at 65,022 Da (Figure 4E), which was conclusively due to the formation of a thio-ester bond between the thio group of 4′-PP and the carboxyl group of L-tryptophan and a concomitant removal of water molecule, despite the mass shift to 65,022 Da, the retention time nearly stayed the same at 16.8 min, since the loaded L-tryptophan residue was too small for the original mass weight 64,836 Da to have notable effect on the retention time of Qui18. In use of the coexpression product of Qui5 and holo-Qui18 as above, yet with D-tryptophan or L-phenylalanine tested, the ion signal stayed unchanged at 64,836 Da (Figure 4C and D), demonstrating no occurrence of any amino acid loading. When holo-Qui18 was used even with the substrate L-tryptophan, the signal remained at 64,836 Da, and the supplementation of Qui5 to the holo-Qui18 turned out to make no difference at all (Figure 4B). The rationale for the failing of the holo-Qui18 supplemented with Qui5 to loading L-tryptophan might be that the *in vitro* condition was not suitable for the interaction of Qui5 with Qui18 and hence Qui5 was not able to assist in the loading even if the coexistence of the two protein, but there was another possibility which could trace back to the earlier stage when Qui18 was expressed in *E. coli* that the Qui18 couldn’t fold correctly without the help of Qui5, the explanation is mainly based on the phenomenon referred to above that much more soluble Qui18 could be derived from the coexpression strain containing pJTU5906 than from the strain containing pJTU5905 for the expression of *qui18* alone. It might be that Qui5 coexpression with Qui18 *in vivo* helped Qui18 fold correctly immediately after the translation of *qui18* and simultaneously Qui5 would bind to Qui18 tightly *in vivo*. In sum, Qui18 recognized L-tryptophan as its substrate and its function depended on the association with Qui5. The association may take place soon after the translation of Qui18.

### Hydroxylation Activity Measurement of Qui15

**Figure 5:**
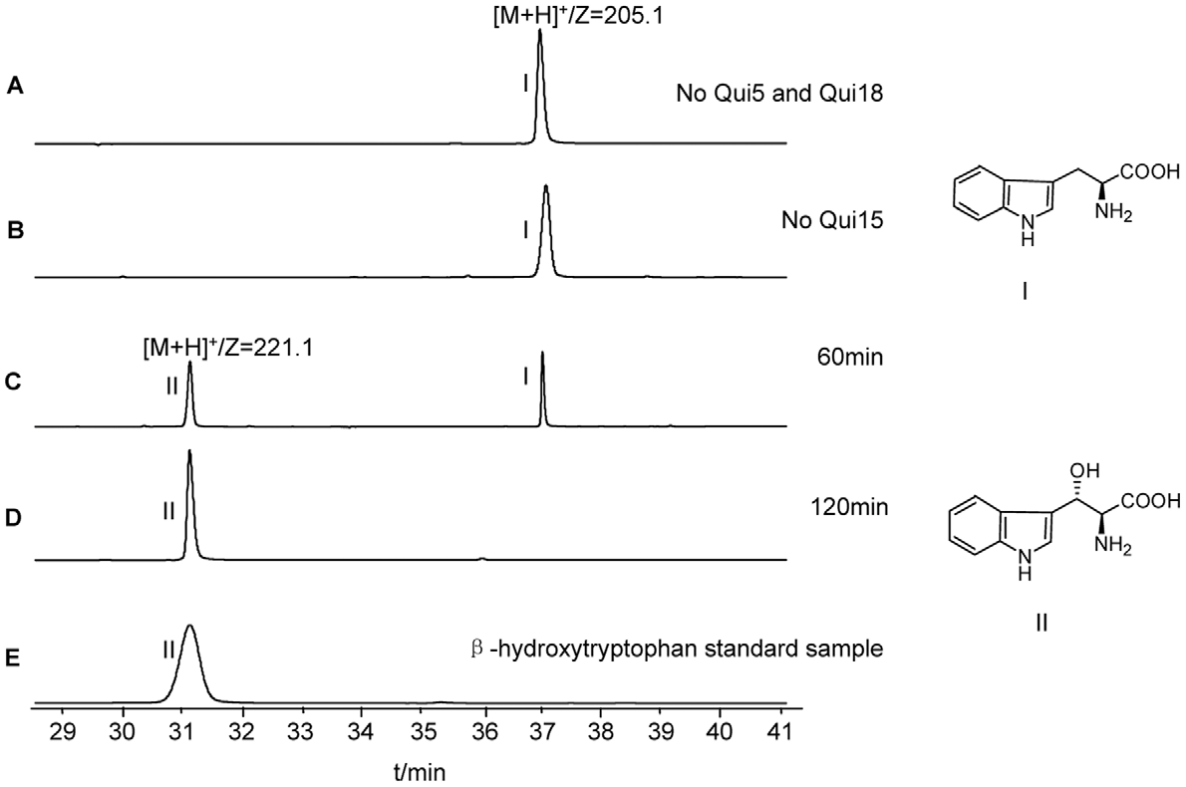
LC-MS analysis of the hydroxylation activity of Qui15. Before the hydroxylation reaction, all the samples were incubated with coexpression of Qui5 and holo-Qui18 for 2 hours to ensure the loading of L-tryptophan other than the negative control of (A). (A) L-tryptophan wasn’t incubated with coexpression of Qui5 and holo-Qui18 and hence all L-tryptophan was free instead of the loaded form; (B) L-tryptophan was incubated with coexpression of Qui5 and holo-Qui18 form but then no Qui15 was supplemented; (C) After the incubation of L-tryptophan and coexpression of Qui5 and holo-Qui18 for 2 hours, Qui15 was supplemented and an additional 60 min incubation was adopted; (D) the same as (C), except the additional incubation time was 120 min; (e) (2*S*,3*S*) β-hydroxytryptophan standard sample. The peak I is L-tryptophan and the peak II is β-hydroxytryptophan.

It has been illuminated that in the biosynthesis of novobiocin, clorobiocin, simocyclinone D8 and vancomycin, the loading of L-tyrosine was a prerequisite for the cytochrome P450 catalyzed β-hydroxylation of L-tyrosine [17] and similar sequential events also happened to the bioxynthesis of nikkomycin, but in which the loaded and β-hydroxylated amino acid was histidine [18]. The above reports have intrigued us to investigate whether the similar case would hold true for echinomycin biosynthetic pathway. Qui15 was a heme-dependent protein, presenting a brownish color and had a molecular weight of about 46 kD per subunit (Figure S4). According to the previous knowledge about β-carbon hydroxylation of amino acids, the reaction could occur only if the putative substrate amino acid was loaded onto NRPS protein. Therefore, before the hydroxylation reaction, L-tryptophan loading reaction proceeded for 2 hours, and then all the necessary factors for hydroxylation were supplemented. To make a reference, the free L-tryptophan was also used as the substrate for Qui15 as a negative control. Two peaks I and II observed in the LC-MS result were assigned as L-tryptophan and its corresponding product β-hydroxytryptophan, emerging respectively at 37 min and 31.2 min (Figure 5). Peak I showed an ion of m/z 205.1, which corresponds to the protonated quasi-molecular ion of L-tryptophan, the fragmentation profile of which is the same as that observed for L-tryptophan standard sample. Peak II had a strong signal at m/z 221.1, which is consistent with the protonated quasi-molecular ion of β-hydroxytryptophan and has the same fragmentation profile as (2*S*,3*S*) β-hydroxytryptophan standard sample (data not shown). In the negative control with free L-tryptophan as the substrate, what was applied to the C_18_ reverse column for LC-MS analysis was the supernatant produced by centrifugation after the addition of trifluoro-acetic acid (TFA), while for the other reaction samples, what was analyzed was the hydrolytic product by KOH from the thioester bond between the carbonyl group of the loaded amino acid and the sulfur atom of the 4′-PP cofactor of Qui18. Therefore, the amount of L-tryptophan observed in the reaction sample reflects how much Qui18 loaded L-tryptophan was not converted by Qui15, while the negative control indicates how much L-tryptophan in all was added to the reaction system. Although, the apparent areas of all peak I were comparable, the factual area of the negative control was much higher than others. The gauge for the ordinate value in the negative control was different from all other samples in the experiment. The negative control showed only Peak I (Figure 5A), which validated the inability of Qui15 to hydroxylate free L-tryptophan. As expected, the sample with the loaded L-tryptophan as the substrate but containing no Qui15, ferredoxin or ferredoxin reductase also presented only Peak I (Figure 5B). The sample with all the necessary proteins and the loaded substrate and incubation for 60 min after the addition of Qui15, both peaks were present (Figure 5C), in comparison, the sample incubated for 120 min only showed peak II, meaning all the loaded L-tryptophan was converted to hydroxytryptophan (Figure 5D). To make a reference, (2*S*,3*S*) β-hydroxytryptophan standard sample was also tested with LC-MS, producing only one peak which was consistent with peak II displayed in the above two reaction samples (Figure 5E). Consequently, the *in vitro* experiments of Qui15 validated the hydroxylation step of Qui18 loaded L-tryptophan catalyzed by Qui15. There is a closely interlocked relationship in terms of enzyme function that MbtH-like protein Qui5 is indispensable for the loading of L-tryptophan by A-PCP didomain NRPS protein Qui18 and only after the loading by Qui18, was cytochrome P450 dependent hydroxylase Qui15 able to utilize L-tryptophan.

### *In vivo* and *in vitro* Analysis of Tryptophan 2,3-dioxygenase Family

In addition to primary metabolism in many organisms, tryptophan 2,3-dioxygenase (TDO) gene also prevailed in the biosynthetic gene cluster of quinomycin family such as triostin, echinomycin, thiocoraline and SW-163. As the hypothesis proposed, the TDO accomplished the oxidative indole ring opening of β-hydroxytryptophan to generate *N*-formyl-β-hydroxykynurenine in these quinomycin biosynthetic pathways. However, the phylogenetic relationship of TDOs was not as close as expected.

**Figure 6:**
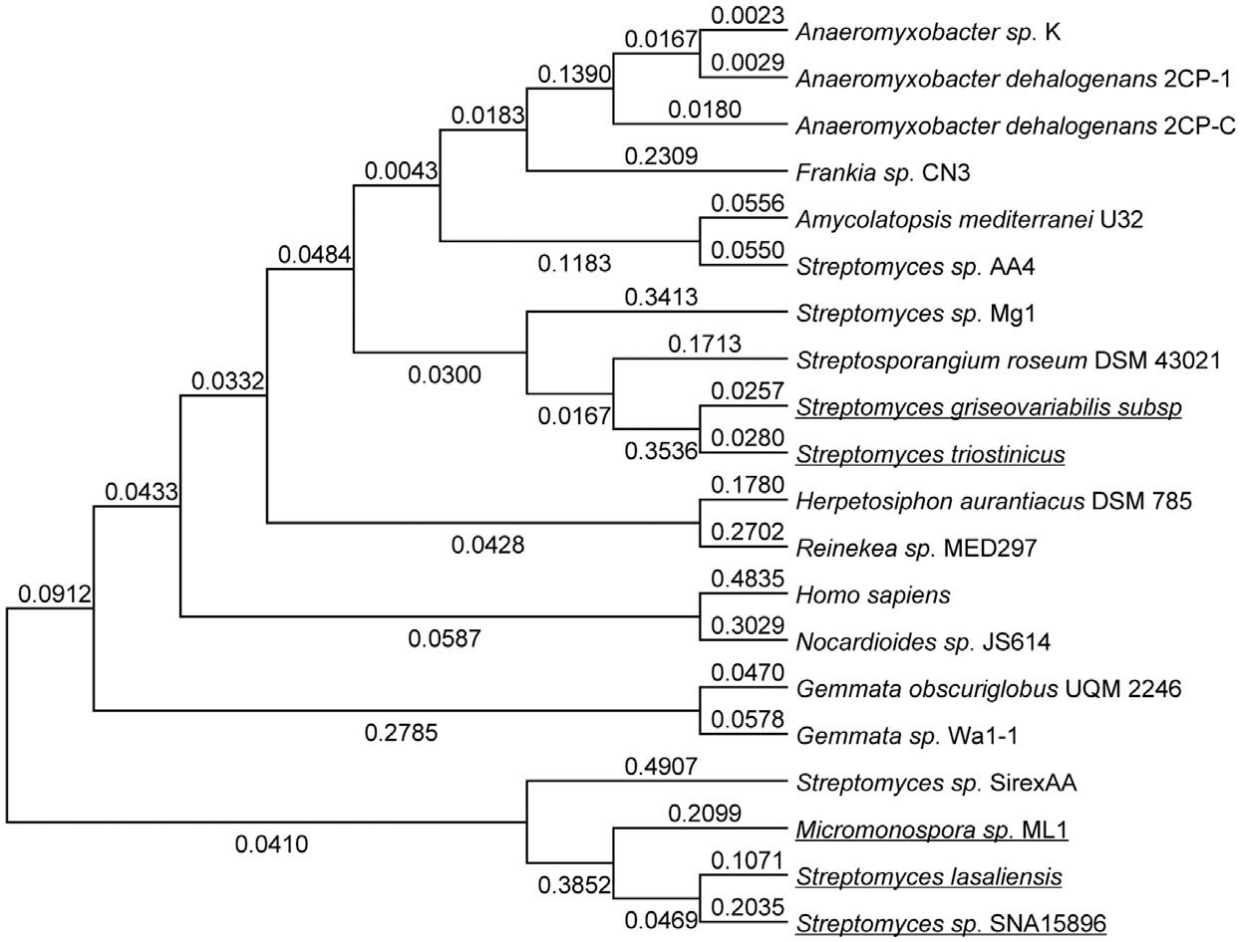
Phylogenetic tree representing evolutionary relationships of the tryptophan 2,3-dioxygenase family. *S. griseovariabilis* is the strain from which the gene cluster was reported by us and it’s an echinomycin producer. *S. triostinicus, S. lasaliensis, micromonospora* sp. ML1 and *S.* sp. SNA15896 respectively produce triostin A, echinomycin, thiocoraline and SW-163. The above five strains are underlined. The apparent evolutionary distances among these family members were shown.

*S. griseovariabilis* and *S. triostinicus* were in the same clade and could be classified in the same group, they respectively produce echinomycin and triostin A as their main products. *S. lasaliensis, micromonospora* sp. ML1 and *S.* sp. SNA15896 were in another group, they respectively produce echinomycin, thiocoraline and SW-163. The phylogenetic tree clearly showed that the two groups are distantly apart (Figure 6). Surprisingly, both *S. griseovariabilis* and *S. lasaliensis* produce echinomycin, yet their TDOs didn’t exhibit close phylogenetic relationship. Further, the alignment of the two nucleotide sequences turned out of no obvious similarity. Even if taken account at amino acid level, the identity was just 46%, still far below the value between other homologs. The rationale for the low conservation of the enzyme may be the splitting of the primary metabolic TDO for the one in the quinomycin biosynthetic pathway to jointly facing the selective pressure during evolution. The primary metabolic TDO might also convert β-hydroxytryptophan. If so, when the TDO gene in the cluster was inactivated, the strain could still generate echinomycin, despite much lower production, as was supported by the data of the following fermentation experiment of the wild and the *qui17* disruption mutant.

**Figure 7:**
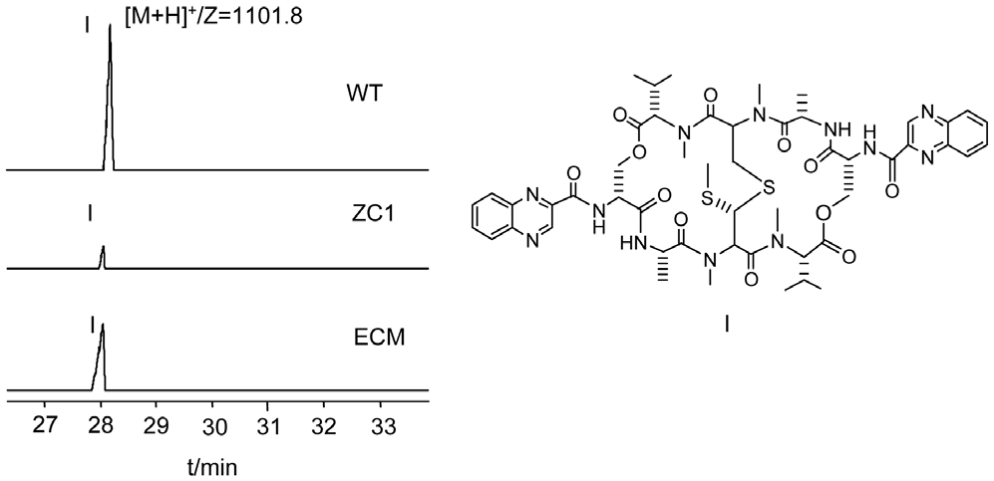
LC-MS comparison of echinomycin standard to the fermentation products from wild-type *S. griseovariabilis* and *qui17*::*aadA* mutant. The peak I is echinomycin; WT: wild-type *S. griseovariabilis*; ZC1: *S. griseovariabilis* ZC1 mutant; ECM: echinomycin standard.

To investigate the extent to which *qui17* deletion would affect the biosynthesis of echinomycin, the gene was disrupted by inserting a spectinomycin resistant gene (*aadA*) cassette, leading to a mutant strain ZC1 (Figure S5), which was verified by PCR (Figure S6). LC-MS was used to analyze the fermentation product of both wild type and ZC1 under the same growth condition. Both wild type and ZC1 presented a peak at 28 min, as is correspondent to the retention time displayed by echinomycin standard (Figure 7), the peak at 28 min generated an ion at m/z 1101.8, which is consistent with the protonated quasi-molecular ion of echinomycin, hence the peak was assigned as echinomycin and the assignment was further confirmed by the same characteristic optical absorption presented by wild type and ZC1 as echinomycin standard at 235 nm and 325 nm (data not shown).

Although the mutant ZC1 remained able to produce echinomycin, the yield was remarkably lower than that of the wild type, suggesting complementary action by some other gene in the mutant. Previous knowledge about TDO family revealed its prevalence in humans, plants and microorganisms, especially as a key enzyme in tryptophan catabolism pathway, and recently, its existence in antibiotics biosynthesis genes cluster has also been reported in triostin, echinomycin, thiocoraline and SW-163 producer streptomyces. According to the general trend of TDO family members existence in organisms, they tend to be responsible for the tryptophan catabolic primary metabolism [19], while in some unusual cases, their genes could be found in gene clusters responsible for secondary metabolism [20]. Therefore, the complementary action might be attributed to the primary metabolic TDO, since TDO gene might well also exist in the chromosome of *S*. *griseovariabilis* but out of the echinomycin biosynthesis gene cluster. Besides, the primary metabolic TDO acted upon L-tryptophan quite specifically, yet it might also recognize and convert β-carbon hydroxylated L-tryptophan at far lower efficiency, leading to the continuation of echinomycin production in the *qui17* disrupted mutant ZC1 at a poor yield.

According to the present hypothesis the hydroxylated tryptophan would be released from the didomain NRPS protein Qui18 by a Type II thioesterase Qui14 to give a free (2*S*,3*S*) β-hydroxytryptophan which would then be catalyzed by TDO Qui17. The conclusion was based on a feeding experiment by kenji watanabe designed to identify (2*S*,3*S*) β-hydroxytryptophan as the intermediate in which the deuterium labeled (2*S*,3*S*) β-hydroxytryptophan and (2*S*,3*R*) β-hydroxytryptophan were synthesized chemically to feed *S. lasaliensis* and at last radioactivity was detected in the final product echinomycin in the case of feeding with (2*S*,3*S*) β-hydroxytryptophan [8]. However, the conclusion went beyond our previous knowledge about TDO that TDO owned such a stringent substrate specificity that it could convert almost only L-tryptophan and couldn’t nearly tolerate any structural analog of L-tryptophan except 6-fluoro-tryptophan [19], [21]. To date, no data has been reported whether TDO could utilize β-hydroxytryptophan, which makes it much more interesting to investigate whether the TDO Qui17 in our echinomycin biosynthesis pathway convert β-hydroxytryptophan. Because of the low amount of β-hydroxytryptophan derived from the *in vitro* reaction of Qui15, we have to prepare adequate β-hydroxytryptophan with chemical method. Following the method of kenji watanabe, we got (2*S*,3*S*) β-hydroxytryptophan through chemical synthesis as the substrate of the *in vitro* experiment [8]. The compound was confirmed by ^1^H NMR spectroscopy (400 MHz, Bruker) and MS to be (2*S*,3*S*) β-hydroxytryptophan. ^1^H NMR (D_2_O, 400 MHz): 4.08 (dd, J = 3.6 Hz, 1H), 5.39 (dd, J = 3.6 Hz, 1H), 6.22 (br, 1H), 7.01–7.05 (m, 1H), 7.10–7.14 (m, 1H), 7.34 (s, 1H), 7.40 (d, J = 8.0 Hz, 1H), 7.59 (d, J = 7.2 Hz, 1H), 8.12 (br, 2H), 11.14 (br, 1H), 13.70 (r, 1H) (Figure S8).

To corroborate the ability of the putative TDO Qui17 to catalyze (2*S*,3*S*) β-hydroxytryptophan, the protein was heterologously expressed and purified from *E. coli*. The calculated molecular weight of His-tagged Qui17 subunit is 30.1 kD, which is consistent with that observed by SDS-PAGE (Figure S7). From the results of size exclusion chromatography, Qui17 is a tetramer, characteristic of TDO family (data not shown). Upon purification, Qui17 presented a dark brown color, which is attributable to its cofactor heme and is another feature of TDO family.

**Figure 8:**
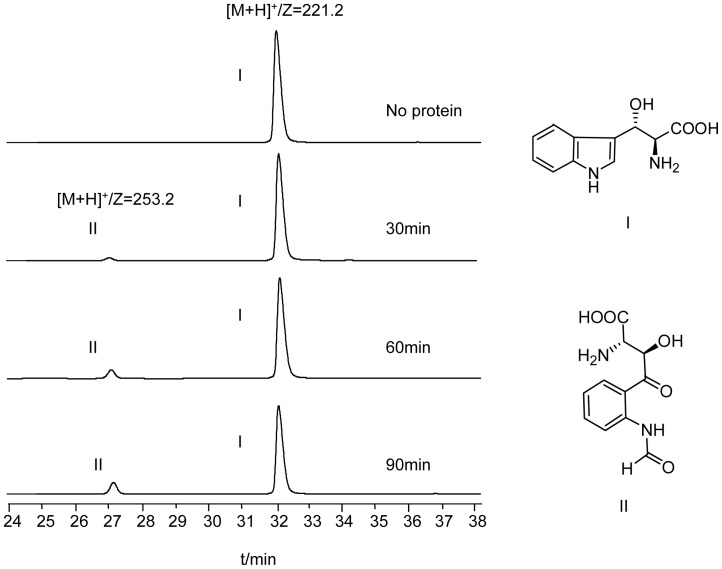
LC-MS analysis of the reaction catalyzed by Qui17. ‘No protein’ represents the sample of negative control lacking Qui17.The reaction was terminated after incubation of 30 min, 60 min, 90 min respectively. The peaks I and II are (2*S*,3*S*) β-hydroxytryptophan and (2*S*,3*R*) *N*-formyl-β-hydroxykynurenine. The chemical structure of each compound is shown.

The activity of Qui17 to oxidize (2*S*,3*S*) β-hydroxytryptophan (HT) to β-hydroxy-*N*-formylkynurenine (HFK) was confirmed by LC-MS. The LC-MS traces show the time-dependent conversion of (2*S*,3*S*) β-hydroxytryptophan to β-hydroxy-N-formylkynurenine (HFK) by Qui17 during a period of 90 min with time interval 30 min (Figure 8). the peak appearing at 32 min showed an ion of m/z 221.2, which corresponds to the protonated quasi-molecular ion of HT and another peak appearing at 27.1 min showed an ion of m/z 253.2, which was assigned to the protonated quasi-molecular ion of the product HFK converted from HT. when no Qui17 was added, the substrate HT was unconsumed all the time, but the formation of HFK was observed with Qui17 added in. even if the yield of HFK was constantly low compared to the amount of the substrate HT, it could be still found that amount of the product increased with time. The yield at 60 min scrambled to nearly threefold of that at 30 min, but from 60 min to 90 min, the yield increased only a little with a large amount of substrate HT left. The large quantity of remnant HT might be due to the stepwise inactivation of Qui17 during 90 min. Qui17, the family member of TDO, depended on heme to realize electron transfer, and heme is readily oxidized from its ferrous active form to ferric inactive form, though ascorbic acid and methylene blue was added to prevent its oxidation. Together with the previous feeding experiment by Kenji Watanabe, the enzymatic assay supported (2*S*,3*S*) β-hydroxytryptophan as the substrate of the TDO and thus an intermediate of the echinomycin biosynthetic pathway.

### Conclusion

The gene cluster in *S. griseovariabilis* reported by this paper has high homology same arrangement with that from *S. triostinicus*. *In vitro* study about the function of Qui5, Qui15, Qui17 and Qui18 confirmed that echinomycin biosynthesis pathway, which starts with the loading of L-tryptophan by an A-PCP didomain NRPS protein Qui18 with the assistance of a MbtH-like protein Qui5, then the loaded L-tryptophan is hydroxylated by cytochrome P450 dependent hydroxylase Qui15. Although, the release of the hydroxytryptophan from Qui18 by the thioesterase Qui14 wasn’t studied in current work, we managed to synthesize the putative hydrolytic product (2*S*,3*S*) β-hydroxytryptophan, which then proved to be converted by TDO Qui17 to form *N*-formyl-β-hydroxykynurenine. The targeted disruption of *qui17* didn’t abolish the production of echinomycin, though lowering its yield apparently, which suggested that the TDO in the primary metabolic pathway might complement Qui17 functionally to some extent. But this is to be confirmed. These data serve well to support the previous hypothesis on echinomycin biosynthesis and pave the way for the investigation of other puzzling points about the biosynthesis of the aromatic precursor QXC.

## Materials and Methods

### Culture Techniques and Genetic Manipulation

*S. griseovariabilis* subsp. bandungensis subsp. nov was cultivated with SFM agar plate at 30°C to collect spores, with YEME culture medium to produce mycelium. Isolation of total DNA and conjugation assay were carried out according to Kieser et al [22]. Operations on *E. coli* were referred to Sambrook et al [23]. Restriction enzymes, T4 DNA ligase, KOD polymerase and alkaline phosphatase were purchased from MBI Fermentas.

### Construction of *S. griseovariabilis* subsp. bandungensis subsp. nov Genomic Library

CopyControl™ Fosmid Library Production Kit (Epicenter, Madison, WI, USA) was directly used for the genomic library construction. High-molecular-weight chromosomal DNA was derived by fracturing total DNA with mechanical force and repaired to blunt end with specific enzyme provided in the kit. Then the end-repaired DNA was fractionated through low-melting temperature agarose in pulsed field [24]. DNA fragment of about 36 kb was excised from the agarose gel and processed with agarase. The resolved DNA was ligated to the vector pCC1FOS (Table S2). Packaging and transduction into EPI300-T1R plating cells was attained with λ-packaging mixes that were offered in the kit (Table S1) [24]. The chloramphenicol resistant colonies were picked one by one and inoculated in LB (100 mL) that contained chloramphenicol in each well of 14 96-well plates. After incubation at 37°C for 20 hr on shaker, 50% glycerol (100 mL) was added into each well for storage at −80°C.

### PCR Primers and DNA Probes

ecm1 and trsA encode AMP-binding ligases respectively in *S. lasaliensis* and *S. triostinicus* which were proposed to activate QXC before its loading onto PCP. trsA and ecm1 have high identity and hence their concensus could be used to design highly conserved primers Adef and Ader for screening the gene cluster among the genomic library. PCR product of about 600 bp was derived after 30 cycles of reactions with the total DNA of *S. griseovariabilis* subsp. bandungensis subsp. nov as template and resolved by agarose gel (0.8%) electrophoresis. DNA Gel Extraction Kit (V-gene Biotechnology Ltd.) was used to retrieve the targeted 600 bp band, which was subsequently cloned into pMD18-T vector (TaKaRa, Dalian, China) for sequencing. The high sequence similarity of the PCR product to *ecm1* (75%) and *trsA* (97%) validated the pair of primers for searching the genomic library for the gene cluster in question. the cells in the same row of 96-well plate were mixed together and inoculated in chloramphenicol containing LB. After 12 hr of culture at 37°C, plasmids were extracted to be used as template for PCR with the pair of primers qui16f and qui16r. The expectant 600 bp product emerged from two samples which correspond to two rows of the 14 96-well plates, subsequently, cells in each well of the two rows was inoculated individually to supply the template for PCR. In the same way, two clones containing the AMP-binding ligase gene were identified.

### Sequence Analysis

Gene cluster was sequenced at Shanghai Major Biotechnology Ltd. (Shanghai, China), and multiple sequence alignment was performed with BioEdit 7.0. Open-reading frames (ORFs) and ribosome binding sites (RBS) were predicted through Frame-Plot 3.0. Similarity comparisons of nucleotide or amino acid sequences against public databases were done by using the BLAST program on the NCBI website [25].

### Targeted Disruption of *qui17*

The disruption of ecm11 by spectinomycin resistance gene aadA was realized through PCR targeting technique (Table S3) [26]. The resistance cassette of about 1.4 kb was obtained by PCR with the template pIJ778 and a pair of primers qui17dF and qui17dR. The resulting fragment was introduced into competent *E. coli* BW25113/pIJ790/K311 (Table S1) [25] by electroporation to replace a 400 bp fragment sited in the *qui17* via double crossover, yielding the recombinant plasmid pJTU5901 (Table S2). The clone containing pJTU5901 was screened by PCR with primers qui17dF and qui17dR (Table S3). pJTU5901 was introduced into *E. coli* ET12567 which could be used to transfer pJTU5901 into *S. griseovariabilis* subsp. bandungensis subsp. nov by conjugation (Table S1). Putative double-crossover strains were confirmed by PCR with the primers of Exqui17F and Exqui17R (Table S3).

### Production and Analysis of Echinomycin

For LC–MS analysis, strains were cultured on GAUZE’s Medium NO.1 for a week, the spore was inoculated to the seed culture media (glucose 1%, maize fermentation liquor 1.5%, tryptone 0.5%, NaCl 0.5%, (NH_4_)_2_SO_4_ 0.5%, CaCO_3_ 0.5%, pH 7.2–7.4), incubated on the shaker at 110 rpm and 29°C for 28 hr. The mycelium suspension was inoculated to the fermentation culture (glucose 1.5%, maize fermentation liquor 1%, soybean powder 2%, starch 1.5%, beef extract 0.35%, NaCl 0.5%, (NH_4_)_2_SO_4_ 0.5%, CaCO_3_ 0.5%, MgSO_4_ 0.01%, FeSO_4_^.^7H_2_O 0.01%, pH 7.2–7.4) media at the ratio of 8 to 100 at 32°C for 96 hr. Centrifuge the culture liquid, extract the supernatant with ethyl acetate and lixiviate the sediment with 60% acetone. The two kinds of extractant were conflated together and concentrated *in vacuo* to present an oily brownish residue which was applied to C_18_ reverse-phase column (5.0×250 mm, 5 µm). Samples were fractionated on a linear gradient of 45–95% CH_3_CN (v/v) in H_2_O supplemented with 0.1% (v/v) formic acid during 55 min at room temperature and flow rate was 0.2 mL/min. Elution was monitored with a photodiode array detector at 280 nm and electrospray ionization (ESI) MS analysis was carried out in the positive mode.

### Construction of pET28-a(+) Derived Vector pCT28

The construction of pCT28 was achieved through targeted mutagenesis on three sites of pET-28a. Three pairs of primers were used in PCR with the template of plasmid pET-28a. First, the primer pair ct1 was designed to remove the original *Xba*I site and meanwhile introduce a *Kpn*I site (which was not existent in pET-28a). The PCR product was digested with *Kpn*I and processed with T4 DNA ligase so that the two ends of the linear product could be connected to form a circular plasmid, the ligation product was transformed into DH10b and the positive clone pJTU5903 was picked out, plasmid pJTU5903 was used as the template for the next PCR mutagensis step. Second, the primer pair of ct2 and another pair of ct3 were used respectively to get the larger and smaller PCR products, both of which had *Xba*I and *Spe*I site respectively at two ends, thus a new *Xba*I site and a *Spe*I site would be introduced in pCT28 accordingly closely upstream the *Bgl*II site and downstream the T_7_ terminator of pET-28a. The two kinds of products were both digested with *Xba*I and *Spe*I and then ligated together by T4 DNA ligase. The ligation product was transformed into DH10b, and the positive transformant with pCT28 was screened, which have all the necessary expression elements and MCS between *Xba*I and *Spe*I site (Table S2).

### Preparation of *qui5, qui15, qui17*and *qui18* Expression Constructs and Coexpression Construct of *qui5* and *qui18*

For construction of *qui5, qui15, qui17* and *qui18* expression plasmids, the four genes were amplified from fosmid K311 by PCR respectively with the primers Exqui5, Exqui15, Exqui17and Exqui18 (Table S3). PCR was carried out with high fidelity DNA polymerase (KOD-plus) and the products were gel-purified, digested with appropriate enzymes and cloned into corresponding restriction sites of pET-28a(+) vector (Novagen) (for construction of expression plasmids of *qui15* and *qui17*) and pCT28 (for construction of expression plasmids of *qui5* and *qui18*). The resulting plasmids, pJTU5902 (for expression of *qui17*), pJTU5904 (for expression of *qui5*), pJTU5905 (for expression of *qui18*) and pJTU5907 (for expression of *qui15*) were transformed into *E. coli* DH10B for sequencing confirmation and into *E. coli* BL21 (DE3) pLysE (Novagen) for expression (Table S2). For construction of coexpression plasmid of *qui5* and *qui18*, pJTU5905 was digested with *Xba*I and *Spe*I, generating a fragment containing *qui*18, pJTU5904 was digested with *Spe*I and dephosphoralated with FastAP, *qui18* containing fragment is cloned into the processed pJTU5904, the resulting plasmid pJTU5906 (for coexpression of *qui5* and *qui18*) was transformed into *E. coli* DH10B for sequencing confirmation and into *E. coli* BL21 (DE3) pLysE (Novagen) for expression (Table S2). For getting holo-Qui18, the chloramphenicol resistant plasmid pSV20 (with *Bacillus subtills* phosphopantetheinyl transferase gene, abbreviated as sfp) was co-transformed into *E. coli* BL21 (DE3) (the chloramphenicol resistant plasmid pLysE was missing by relaxation) with pJTU5905 or pJTU5906 [27].

### Expression and Purification of His-tagged Qui5, Qui15, Qui17 and Qui18

*E. coli* BL21 (DE3) pLysE cells that carried pJTU5902, pJTU5904, pJTU5905, pJTU5906 and pJTU5907 and *E. coli* BL21 (DE3) cells that carried both pSV20 and pJTU5905 or pJTU5906 were grown overnight in LB media that was supplemented with chloramphenicol and kanamycin (34 mg/mL and 50 mg/mL respectively). The seed cultures (10 mL) were used to inoculate 1 L production cultures of 2×YT with the corresponding antibiotics. The cells were grown at 37°C to an optical density at 600 nm (OD_600_) of 0.7 and then induced with isopropyl-β-D-thiogalactopyranoside (IPTG, finalconcentration, 1 mM). The cultures were then grown for an additional 12 hr at 16°C. After centrifugation, the cells were resuspended in binding buffer (40 mL, 50 mM Tris-HCl 0.15 M NaCl, pH 8.0) and lysed by FrenchPress. After centrifugation (16000 g for 45 min at 4°C), the supernatant was applied to HiTrap HP column (GE Healthcare) and purified by an Amershan Biosciences FPLC (GE Healthcare), by eluting with elution buffer (50 mM Tris-HCl, 1 M imidazole, and 0.15 M NaCl, pH 8.0) in linear gradient. The purified His-tagged Qui5, Qui17 and Qui18 and copurified His-tagged Qui5 and holo or apo-Qui18 was desalted and concentrated with Millipore Amicon Ultra Centrifugal Filter Units and stored in Tris-HCl buffer (50 mM, pH8.0) with glycerol (20%) at −80°C. The expression and purification of His-tagged Qui17 was analyzed by 12% SDS-PAGE and Qui5, apo or holo-Qui18 and copurification of Qui5 and apo or holo-Qui18 by 15% SDS-PAGE and protein concentrations were determined using the Bradford Protein Assay Kit (Bio-Rad).

### *In vitro* Analysis of Qui18

Loading of amino acid onto holo-Qui18 catalyzed by itself was performed in 50 µL reaction system containing 50 mM Tris-HCl (pH 8.0), 10 mM MgCl_2_, 2 mM DTT, 5 mM ATP, 5 mM substrate (L-tryptophan, D-tryptophan or L-phenylalanine), coexpression product of Qui5 and holo-Qui18 (38 µM Qui5 and 125 µM holo-Qui18) or holo-Qui18 alone (125 µM) with or without additional Qui5 (93 µM). Reactions were initiated by addition of the coexpression product, incubated at 27°C for 2 hr, and quenched by flash freezing at −80°C. The identities of products were determined by Q-TOF-MS.

### *In vitro* Analysis of Qui15

After the Qui18 mediated L-tryptophan loading reaction proceeds for 2 hr, NADPH (2 mM), spinach ferredoxin (Sigma, 5 µM), ferredoxin reductase (Sigma, 0.1 U) and Qui15 (50 µM) were added into the original 50 µL reaction system, the volume of the reaction system for the hydroxylation was 100 µL. The reaction was started by the addition of Qui15 and occurred at 25°C. After 2 hr of incubation, the reaction was quenched with 1 mL 10% trichloro acetic acid (TCA) and then centrifuged. The pellet was washed with 1 mL water for three times. 100 µL 0.1 M KOH was used to redissolve the pellet, after the system was clarified, it was incubated at 60°C for 10 min. the protein was reprecipitated by the addition of 5 µL 50% TFA. After centrifugation, the pellet was removed and the supernatant was analyzed by LC-MS with a C_18_ reverse-phase column (5.0×250 mm, 5 µm) at the flow rate of 0.2 mL/min and the monitor wave length of 280 nm. The elution was done with solvent A (H_2_O, 0.1% TFA) and solvent B (acetonitrile). The elution profile adopted linear gradient in which the concentration of solvent B rose from 10% to 50% during 50 min. Particularly, a negative control was designed in which L-tryptophan was added into the hydroxylation reaction system catalyzed by Qui15 without the preceding loading reaction catalyzed by Qui18 and other conditions were exactly the same. After incubation for 2 hr, 5 µL 50% TFA was added into the negative control reaction system, the protein was precipitated and the supernatant was analyzed by LC-MS with the above method.

### *In vitro* Analysis of Qui17

Before doing the assay, the compound (2*S*,3*S*) β-hydroxytryptophan was chemically synthesized for test as substrate, following the method in a paper by Kenji Watanabe yet without the deuterium label [8]. The assay of Qui17 was carried out at 27°C for 2 hr in a total volume of 50 µL that contained Tris-HCl buffer (50 mM, pH 8.0), (2*S*,3*S*) β-hydroxytryptophan (9 mM), ascorbic acid (6 mM), methylene blue (4 µM) and His-tagged Qui17 (3 µM). The reactions were quenched by the addition of trichloroacetic acid. The reaction mixture was centrifuged at 13000 rpm for 5 min. The supernatant is filtered before analyzed by LC-MS as described above.

## Supporting Information

Figure S1 PCR screening of the genomic library for echinomycin biosynthesis gene cluster.
(DOC)

Figure S2 SDS-PAGE analysis of Qui5 and Qui18.
(DOC)

Figure S3 Measurement of molecular weight of His6-tagged Qui5 and Qui18 complex by size exclusion chromatography.
(DOC)

Figure S4 SDS-PAGE analysis of Qui15.
(DOC)

Figure S5 Illustration of the targeted disruption of qui17 through the technique of PCR targeting.
(DOC)

Figure S6 Confirmation of the *S. griseovariabilis* ZC1 mutant by PCR.
(DOC)

Figure S7 SDS-PAGE analysis of Qui17.
(DOC)

Figure S8 1H-NMR data of the chemically synthesized (2*S*,3*S*) β-hydroxytryptophan.
(DOC)

Table S1 Strains used in this study.
(DOC)

Table S2 Plasmids and fosmid used in this study.
(DOC)

Table S3 Primers used in this study.
(DOC)
