# Supplementary material for: In Vitro Characterization of Echinomycin Biosynthesis: Formation and Hydroxylation of L-Tryptophanyl-S-Enzyme and Oxidation of (2S,3S) β-Hydroxytryptophan
Source: PLoS One. 2013 Feb 21;8(2):e56772. doi: 10.1371/journal.pone.0056772 (PMC3578932; doi:10.1371/journal.pone.0056772)
Supplement: Figure S1 — PCR screening of the genomic library for echinomycin biosynthesis gene cluster. (DOC) [file pone.0056772.s001.doc]

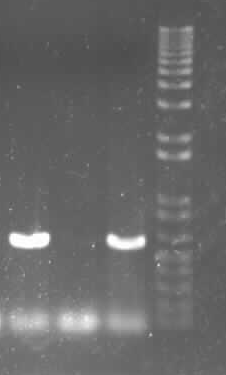


4

3

2

1

600 bp


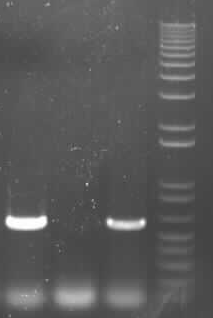


4

3

2

1

600 bp

**A**

**B**

**Figure S1.** PCR screening of the genomic library for echinomycin biosynthesis gene cluster. The primers were adeF and adeR. **A.** Lane 1 and 3 were the products amplified from different templates which were respectively total DNA of *S. griseovariabilis* and the fosmid K111. Lane 2 was a negative control without template and therefore didn’t exhibit the targeted band of 600 bp. **B.** the same as **A**, except for that lane 3 was PCR product amplified from the template A311.
