# Supplementary material for: In Vitro Characterization of Echinomycin Biosynthesis: Formation and Hydroxylation of L-Tryptophanyl-S-Enzyme and Oxidation of (2S,3S) β-Hydroxytryptophan
Source: PLoS One. 2013 Feb 21;8(2):e56772. doi: 10.1371/journal.pone.0056772 (PMC3578932; doi:10.1371/journal.pone.0056772)
Supplement: Figure S2 — SDS-PAGE analysis of Qui5 and Qui18. (DOC) [file pone.0056772.s002.doc]

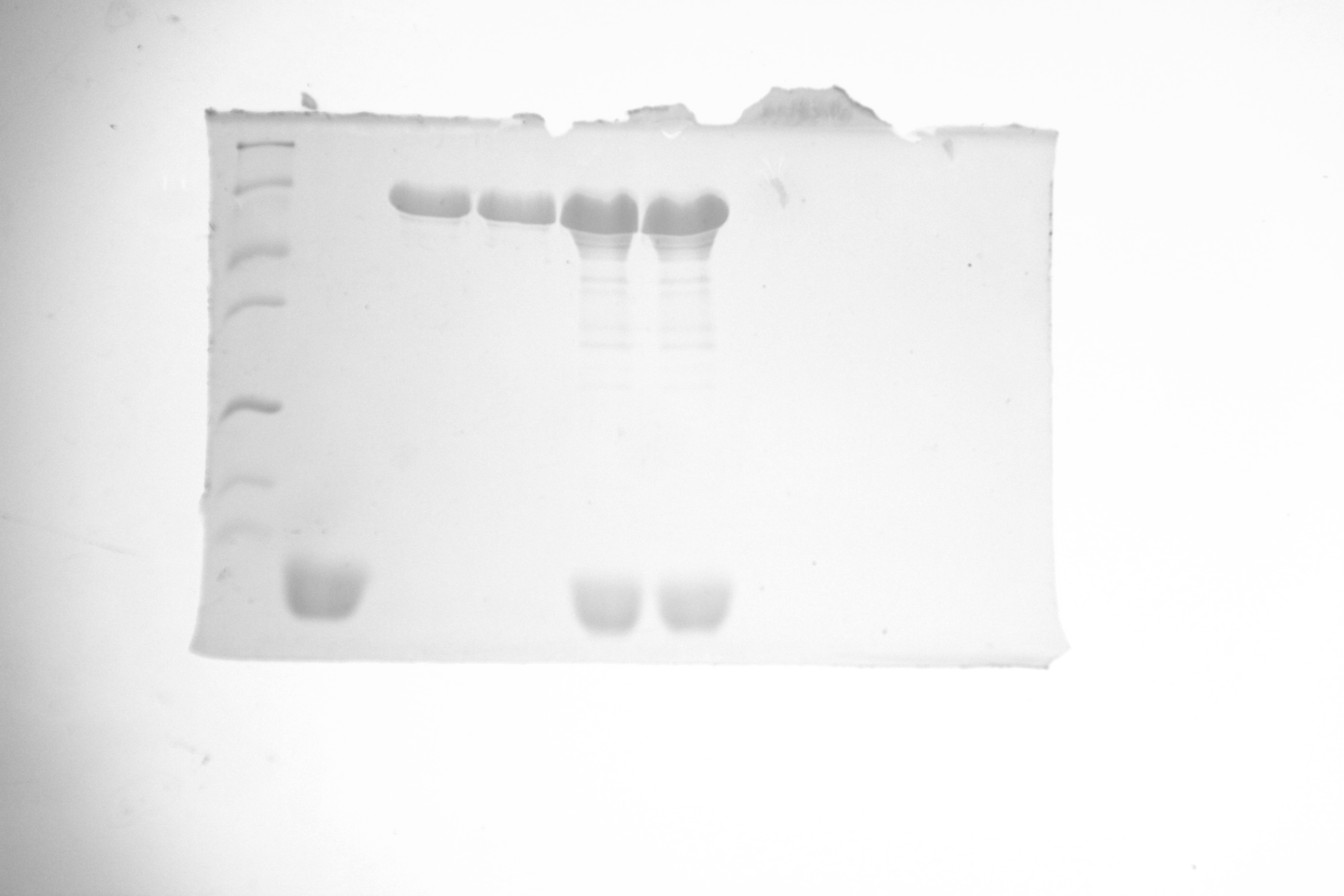


10 kD

64.5 kD

64.8 kD

64.5 kD

10 kD

1

2

3

4

5

6

10 kD

64.8 kD

116.0 kD

66.2 kD

45.0 kD

35.0 kD

25.0 kD

18.4 kD

14.4 kD

**Figure S2.** SDS-PAGE analysis of Qui5 and Qui18. Lane 1. molecular weight markers ; Lane 2: purified His6-tagged Qui5 (M_W_: 10.09 kD); Lane 3: purified His6-tagged apo-Qui18 (M_W_: 64.50 kD); Lane 4: purified His6-tagged holo-Qui18 (M_W_: 64.84 kD); Lane 5: coexpressed and copurified His6-tagged Qui5 and His6-tagged apo-Qui18; Lane 6: coexpressed and copurified His6-tagged Qui5 and His6-tagged holo-Qui18. Before loading, the coexpression sample in Lane 5 and 6 were diluted by 100 fold.
