# Supplementary material for: In Vitro Characterization of Echinomycin Biosynthesis: Formation and Hydroxylation of L-Tryptophanyl-S-Enzyme and Oxidation of (2S,3S) β-Hydroxytryptophan
Source: PLoS One. 2013 Feb 21;8(2):e56772. doi: 10.1371/journal.pone.0056772 (PMC3578932; doi:10.1371/journal.pone.0056772)
Supplement: Figure S3 — Measurement of molecular weight of His6-tagged Qui5 and Qui18 complex by size exclusion chromatography. (DOC) [file pone.0056772.s003.doc]

0

500

1000

1500

mAU

0.0

10.0

20.0

30.0

40.0

50.0

60.0

min

**A**

25.61 min


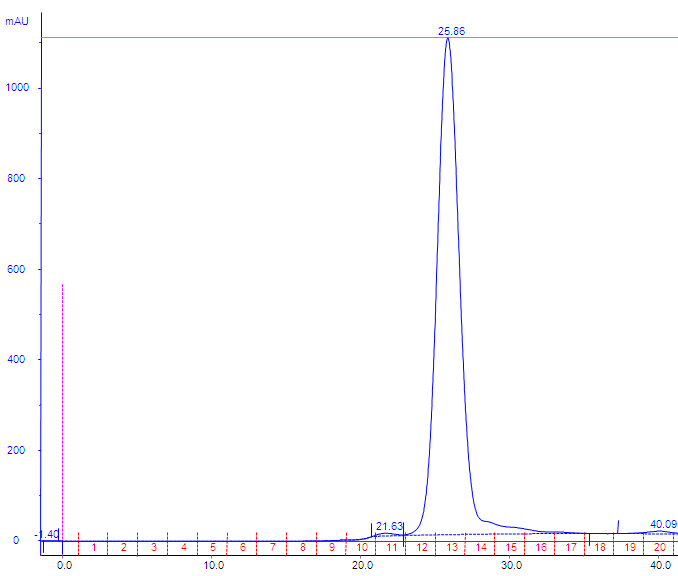


**B**

25.86 min

Aldolase-158 KD


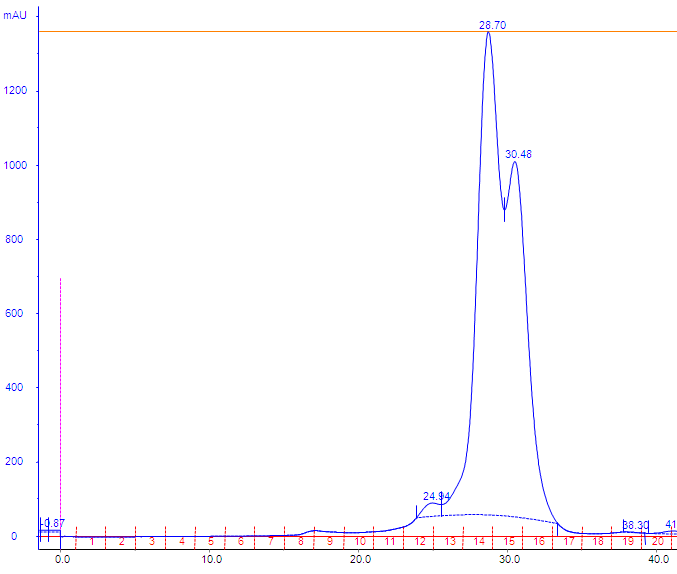


**C**

Conalbumin-75 KD

28.70 min

30.48 min

Ovalbumin-43 KD

**Figure S3.** Measurement of molecular weight of His6-tagged Qui5 and Qui18 complex by size exclusion chromatography. **A.** Size exclusion chromatography analysis of coexpression and copurified His6-tagged Qui5 and Qui18; **B.** size exclusion chromatography analysis of marker aldolase (M_W_: 158 kD); **C.** size exclusion chromatography analysis of markers conalbumin (M_W_: 75 kD) and ovalbumin (M_W_: 43 kD). Qui5 and Qui18 coexpression product present a peak with the retention time very close to that of the marker aldolase at 25.61 min, so the complex of Qui5 and Qui18 has an approximate molecular weight of 160 kD.
