# Supplementary material for: In Vitro Characterization of Echinomycin Biosynthesis: Formation and Hydroxylation of L-Tryptophanyl-S-Enzyme and Oxidation of (2S,3S) β-Hydroxytryptophan
Source: PLoS One. 2013 Feb 21;8(2):e56772. doi: 10.1371/journal.pone.0056772 (PMC3578932; doi:10.1371/journal.pone.0056772)
Supplement: Figure S4 — SDS-PAGE analysis of Qui15. (DOC) [file pone.0056772.s004.doc]

46 kD


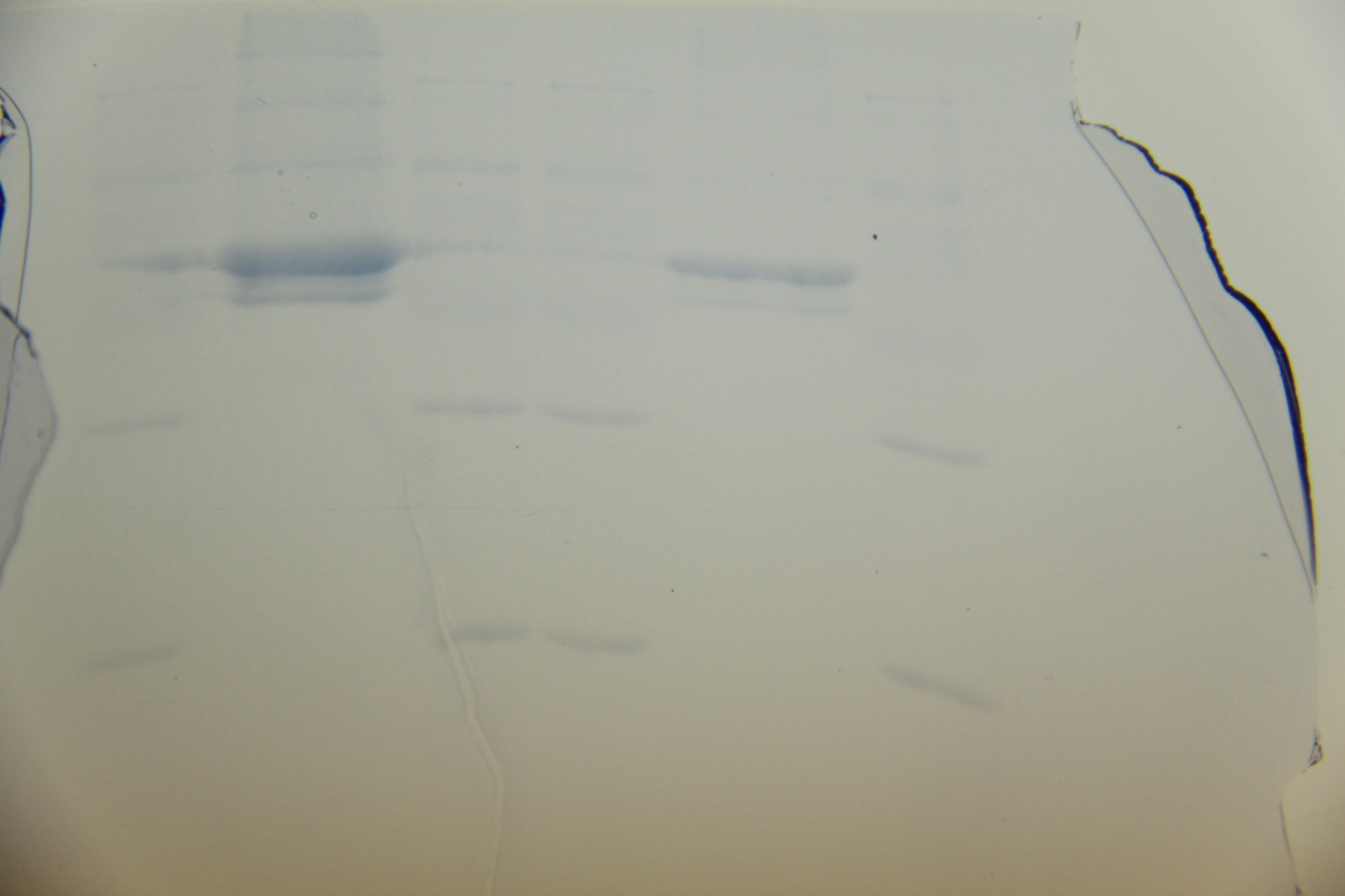


45.0 kD

116 kD

66.2 kD

35.0 kD

25.0 kD

1

2

**Figure S4.** SDS-PAGE analysis of Qui15. Lane 1. molecular weight markers; Lane 2: purified His6-tagged Qui15 (M_W_: 46 kD).
