# Supplementary material for: In Vitro Characterization of Echinomycin Biosynthesis: Formation and Hydroxylation of L-Tryptophanyl-S-Enzyme and Oxidation of (2S,3S) β-Hydroxytryptophan
Source: PLoS One. 2013 Feb 21;8(2):e56772. doi: 10.1371/journal.pone.0056772 (PMC3578932; doi:10.1371/journal.pone.0056772)
Supplement: Figure S5 — Illustration of the targeted disruption of qui17 through the technique of PCR targeting. (DOC) [file pone.0056772.s005.doc]

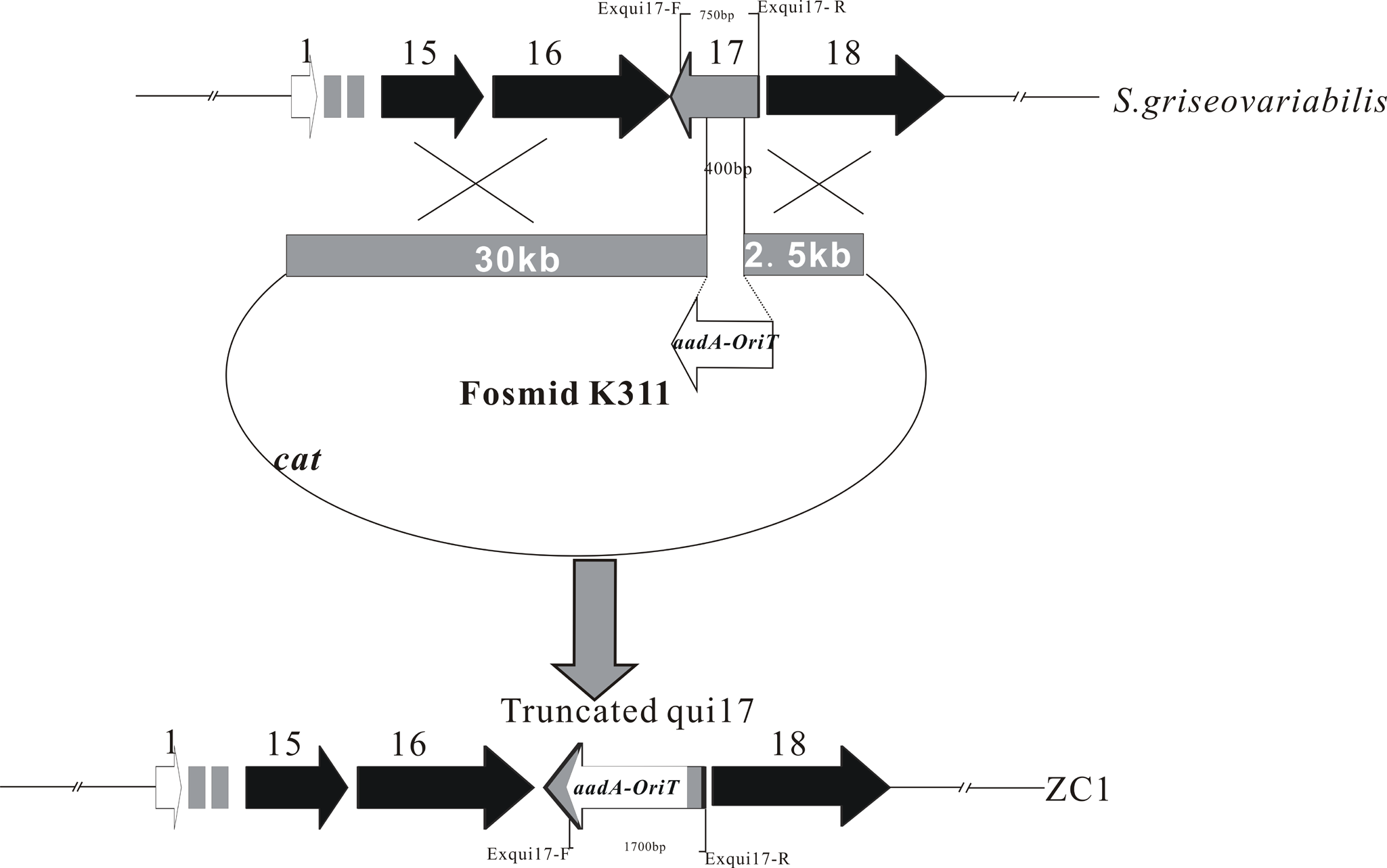


**Figure S5**. Illustration of the targeted disruption of qui17 through the technique of PCR targeting.
