# Supplementary material for: In Vitro Characterization of Echinomycin Biosynthesis: Formation and Hydroxylation of L-Tryptophanyl-S-Enzyme and Oxidation of (2S,3S) β-Hydroxytryptophan
Source: PLoS One. 2013 Feb 21;8(2):e56772. doi: 10.1371/journal.pone.0056772 (PMC3578932; doi:10.1371/journal.pone.0056772)
Supplement: Figure S6 — Confirmation of the S. griseovariabilis ZC1 mutant by PCR. (DOC) [file pone.0056772.s006.doc]

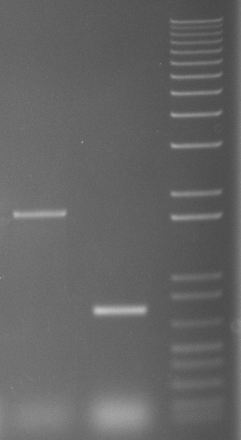


1

2

3

1.75 kb

0.75 kb

**Figure S6**. Confirmation of the *S. griseovariabilis* ZC1 mutant by PCR. Lane 1 shows the PCR product from a clone of the mutant ZC1; Lane 2 shows the PCR product that was obtained from the wild type *S. griseovariabilis*; Lane 3 shows the marker. the pair of primers was Exqui17F and Exqui17R. The wild type and mutant present the bands of 0.75 and 1.70 kb.
