# Supplementary material for: In Vitro Characterization of Echinomycin Biosynthesis: Formation and Hydroxylation of L-Tryptophanyl-S-Enzyme and Oxidation of (2S,3S) β-Hydroxytryptophan
Source: PLoS One. 2013 Feb 21;8(2):e56772. doi: 10.1371/journal.pone.0056772 (PMC3578932; doi:10.1371/journal.pone.0056772)
Supplement: Figure S7 — SDS-PAGE analysis of Qui17. (DOC) [file pone.0056772.s007.doc]

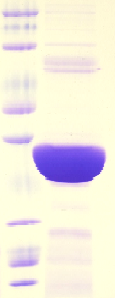


1

2

Qui17

30 kD

116.0 kD

66.2 kD

45.0 kD

35.0 kD

25.0 kD

18.4 kD

14.4 kD

**Figure S7**. SDS-PAGE analysis of Qui17. Lane 1. molecular weight markers ; Lane 2: purified His6-tagged Qui17 (M_W_: 30 kD).
