# Supplementary figures and images for: In Vitro Characterization of Echinomycin Biosynthesis: Formation and Hydroxylation of L-Tryptophanyl-S-Enzyme and Oxidation of (2S,3S) β-Hydroxytryptophan
Source: PLoS One. 2013 Feb 21;8(2):e56772. doi: 10.1371/journal.pone.0056772 (PMC3578932; doi:10.1371/journal.pone.0056772)

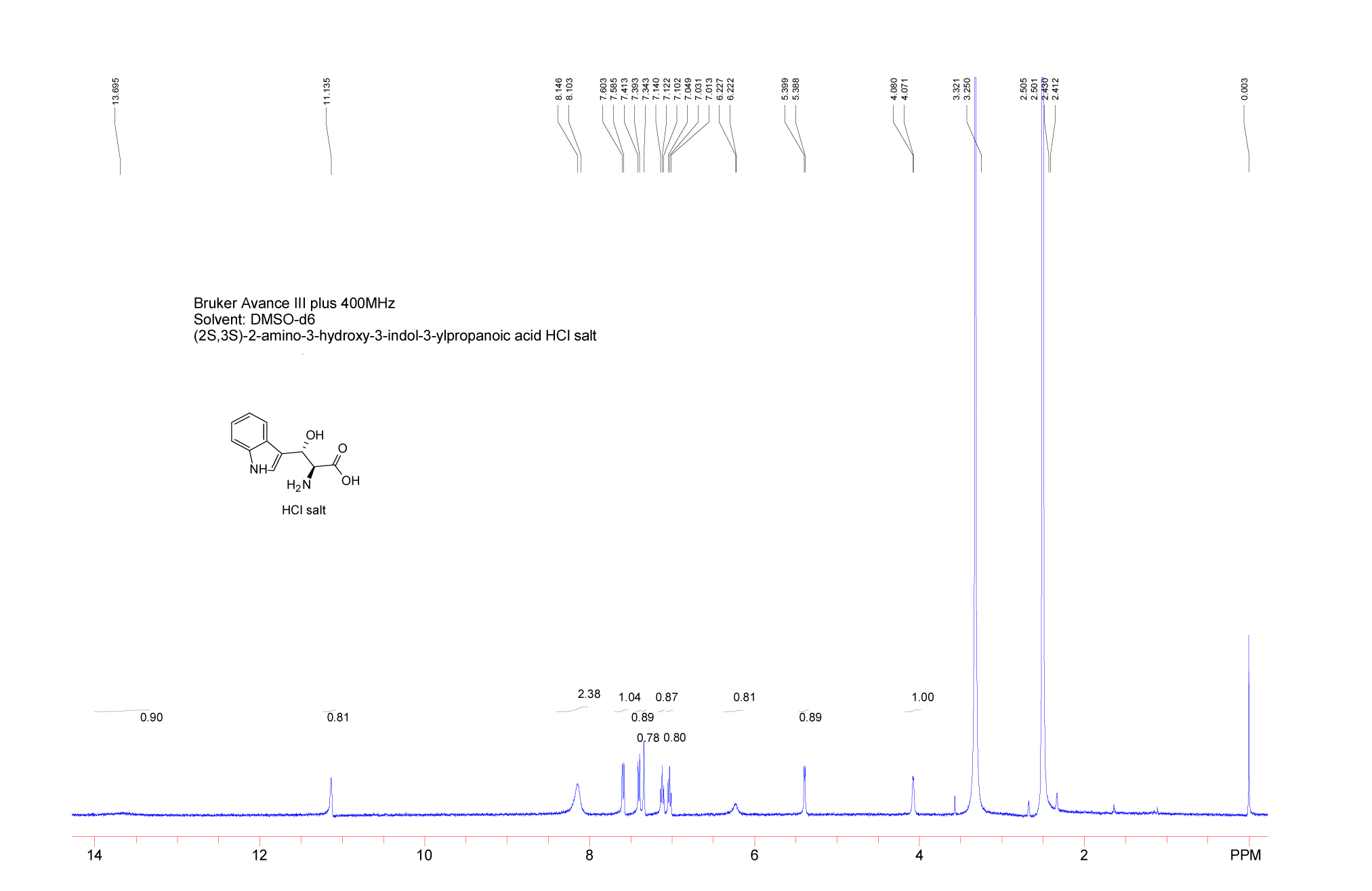


**Figure S8**. 1H-NMR data of the chemically synthesized (2*S*,3*S*) β-hydroxytryptophan.

Supplement: Figure S8 — 1H-NMR data of the chemically synthesized (2S,3S) β-hydroxytryptophan. (DOC) [file pone.0056772.s008.doc]
