# Supplementary material for: In Vitro Characterization of Echinomycin Biosynthesis: Formation and Hydroxylation of L-Tryptophanyl-S-Enzyme and Oxidation of (2S,3S) β-Hydroxytryptophan
Source: PLoS One. 2013 Feb 21;8(2):e56772. doi: 10.1371/journal.pone.0056772 (PMC3578932; doi:10.1371/journal.pone.0056772)
Supplement: Table S1 — Strains used in this study. (DOC) [file pone.0056772.s009.doc]

**Table S1.** Strains used in this study

| **Strain** | **Relevant properties** | **Source/Ref** |
| --- | --- | --- |
| *Streptomyces griseovariabilis* subsp. bandungensis subsp. nov | Producer of echinomycin | South China Agriculture Univ. |
| ZC1 | The mutant of *S. griseovariabilis* subsp. bandungensis subsp. nov with *qui17* disrupted by spectinomycin resistant gene cassette | This study |
| *Escherichia coli* DH10B | F^-^recA lacZΔM15 | GIBCO BRL |
| *E. coli* ET12567/pUZ8002 | Strain that was used for conjugation between *E.coli* and *S. griseovariabilis recF, dam, dcm*, *hsdS*, Cml^r^, Str^r^, Tet^r^, Km^r^ |  |
| *E. coli* EPI300-T1^R^ | F- mcrA Δ(*mrr*-*hsd*RMS-*mcr*BC) 80d *lacZ* M15 *lac*X74 *rec*A1 *end*A1 *ara*D139 (*ara*, *leu*) 7697 *gal*U *gal*K - *rps*L *nup*G *trf*A *ton*A *dhfr* | EPICENTRE Biotechnologies |
| *E. coli* BW25113/pIJ790 | (*oriR101),* (*repA101*(ts)) *araBp-gam-be-exo* Δ(*araD-araB*)*567*,Δ*lacZ4787*(::*rrnB-4*),*lacIp-4000*(lacI^Q^), λ^-^, *rpoS369*(Am), *rph-1*, Δ(*rhaD-rhaB*)568, *hsdR514* | [2] |
| *E. coli* BW25113/pIJ790/K311 | *E. coli* BW25113/pIJ790 derived strain having the cosmid K311 introduced in through transformation | This study |

**SUPPLEMENTAL REFERENCES**

1. Kieser T, Bibb MJ, Chater KF, Butter MJ, Hopwood DA (2000) Practical Streptomyces Genetics: Norwich, UK: John Innes Foundation.
2. Gust B, Challis GL, Fowler K, Kieser T, Chater KF (2003) PCR targeted Streptomyces gene replacement identifies a protein domain needed for biosynthesis of the sesquiterpene soil odor geosmin. Proc Natl Acad Sci U S A 100: 1541-1546.
