# Supplementary material for: In Vitro Characterization of Echinomycin Biosynthesis: Formation and Hydroxylation of L-Tryptophanyl-S-Enzyme and Oxidation of (2S,3S) β-Hydroxytryptophan
Source: PLoS One. 2013 Feb 21;8(2):e56772. doi: 10.1371/journal.pone.0056772 (PMC3578932; doi:10.1371/journal.pone.0056772)
Supplement: Table S2 — Plasmids and fosmid used in this study. (DOC) [file pone.0056772.s010.doc]

**Table S2.** Plasmids and fosmid used in this study

| **Plasmid** | **Relevant properties** | **Source** |
| --- | --- | --- |
| pIJ779 | pBluescript KS(+), aadA, oriT(RK2), FRT sites |  |
| pCC1FOS | Cml^r^, *ori*V, *cos* site, P1 *lox*P site | EPICENTRE Biotech |
| A111 | pCC1FOS derived cosmid with putative echinomycin biosynthesis gene cluster from Streptomyces griseovariabilis subsp. bandungensis subsp. nov | This study |
| K311 | pCC1FOS derived cosmid with putative echinomycin biosynthesis gene cluster from Streptomyces griseovariabilis subsp. bandungensis subsp. nov | This study |
| pJTU5901 | K311 derived cosmid with *qui17* disrupted by a spectinomycin resistant(aadA) cassette together with oriT site | This study |
| pJTU5902 | *qui17* expression vector, PCR product for *qui17* has *Nde*I and *Eco*RI restriction sites at its two ends, which were used for cloning into the corresponding sites of pET-28a(+) | This study |
| pJTU5903 | pET-28a derived vector, the original *Xba*I was substituted with a *Kpn*I site | This study |
| pCT28 | pET28-a derived vector, the original *Xba*I was substituted with a *Kpn*I site, while a new *Xba*I site was introduced proximately upstream *Bgl* II site, and a *Spe*I site was introduced closely downstream T7 transcription terminator | This study |
| pJTU5904 | *qui5* expression vector, PCR product for *qui5* has *Nde*I and *Eco*RI restriction sites at its two ends, which were used for cloning into the corresponding sites of pCT28 | This study |
| pJTU5905 | *qui18* expression vector, PCR product for *qui18* has *Nde*I and *Eco*RI restriction sites at its two ends, which were used for cloning into the corresponding sites of pCT28 | This study |
| pJTU5906 | *qui5* and *18* coexpression vector, pJTU5905 is digested with *Xba*I and *Spe*I, generating a fragment containing *qui*18, pJTU5904 is digested with *Spe*I and dephosphoralated with FastAP, *qui18* containing fragment is cloned into the processed pJTU5904 | This study |
| pJTU5907 | *qui15* expression vector, PCR product for *qui18* has *Nde*I and *Eco*RI restriction sites at its two ends, which were used for cloning into the corresponding sites of pET-28a(+) | This study |

**SUPPLEMENTAL REFERENCES**

1. Gust B, Challis GL, Fowler K, Kieser T, Chater KF (2003) PCR targeted Streptomyces gene replacement identifies a protein domain needed for biosynthesis of the sesquiterpene soil odor geosmin. Proc Natl Acad Sci U S A 100: 1541-1546.
