# Supplementary material for: In Vitro Characterization of Echinomycin Biosynthesis: Formation and Hydroxylation of L-Tryptophanyl-S-Enzyme and Oxidation of (2S,3S) β-Hydroxytryptophan
Source: PLoS One. 2013 Feb 21;8(2):e56772. doi: 10.1371/journal.pone.0056772 (PMC3578932; doi:10.1371/journal.pone.0056772)
Supplement: Table S3 — Primers used in this study. (DOC) [file pone.0056772.s011.doc]

**Table S3.** Primers used in this study

| **Primers** | **Forward (F)** | **Reverse (R)** |
| --- | --- | --- |
| Ade | AGGACGAGTTCGGCGGGTTC | AGACCTTCGCTGATGCCGTACCA |
| qui17d | CAGTCGCACGAGTTGTGGTTCAAGGAGCTGCTCCACGAACTGGACGCCATTCCGGGGATCCGTCGACC | CTTGTTGCCGATCATCCG CTGGACGAGAACGACATGCCGGTACTGCCATGTAGGCTGGAGCTGCTTC |
| Exqui17 | GGGAATTCCATATGAGTAATTCCCATTCCGCCT | CCGGAATTCTACTCGCCGTGCCGTGC |
| Exqui5 | GGGAATTCCATATGAGTATCAACCCGTTCGACG | CCGGAATTCTACCGGTTCATCGCCTCC |
| Exqui18 | GGGAATTCCATATGGGCGATGCAAAGCACA | CCGGAATTCTACGGCTCGTGGTGCGTGC |
| Exqui15 | GGGAATTCCATATGAGCCCGTCGCTCGATCT | CCGCTCGAGCTAGGAGGCGGAGCGCGACT |
| ct1 | CGGGGTACCAATAATTTTGTTTAACTTTAAGAAG | CGGGGTACCGGGGAATTGTTATCCGCT |
| ct2 | CTAGTCTAGAAGATCTCGATCCCGCGAAAT | GGACTAGTCAAAAAACCCCTCAAGACC |
| ct3 | GGACTAGTCTGAAAGGAGGAACTATA | CTAGTCTAGACGATCCTCTACGCCGGAC |
